# Co-Design of a Depression Self-Management Tool for Adolescent and Young Adult Cancer Survivors: User-Centered Design Approach

**DOI:** 10.2196/67175

**Published:** 2025-03-24

**Authors:** Karly M Murphy, Rachel Glock, David Victorson, Madhu Reddy, Sarah A Birken, John M Salsman

**Affiliations:** 1 Department of Psychology East Carolina University Greenville, NC United States; 2 Department of Medical Social Sciences Northwestern University Feinberg School of Medicine Chicago, IL United States; 3 Department of Informatics University of California Irvine Irvine, CA United States; 4 Department of Implementation Science Wake Forest University School of Medicine Winston-Salem, NC United States; 5 Department of Social Sciences and Health Policy Wake Forest University School of Medicine Winston-Salem, NC United States

**Keywords:** adolescent and young adult, cancer survivorship, depressive symptoms, self-management, co-design workshops, user-centered design, thematic analysis, intervention tailoring, digital mental health, evidence-based intervention, digital tools, psychosocial support

## Abstract

**Background:**

Adolescent and young adult (AYA) cancer survivors are more likely to experience elevated depressive symptoms than older survivors and healthy age-matched peers. Despite the elevated risk of depressive symptoms in AYA cancer survivors and the existence of evidence-based interventions to address depression, it is unclear whether AYA cancer survivors can access support services. Digital tools are a potential solution to overcoming barriers to AYA cancer survivors’ unmet needs for psychosocial support, but they have not been tailored to the needs and preferences of this unique population.

**Objective:**

This study engaged AYA cancer survivors and their providers in the concept generation and ideation step of the user-centered design process through online co-design workshops. The goal was to generate concepts and ideas for a digital depression self-management tool tailored to AYA cancer survivors.

**Methods:**

We conducted 5 co-design workshops—4 with AYA cancer survivors and 1 with providers who serve them. Participants were asked to provide feedback on an existing digital mindfulness course using an “I like, I wish, I wonder” framework. Then, participants were asked “How might we...” questions focused on brainstorming ideas for how the digital tool might work. Participants brainstormed responses independently and then worked as a group to categorize and expand on their ideas. Co-design workshops were autotranscribed using Webex (Cisco) software. Transcripts underwent thematic analysis with additional context provided by the products created during the workshop.

**Results:**

Eight AYA cancer survivors (aged 15-37 years) and 4 providers (2 oncologists and 2 social workers) participated in co-design workshops. We identified 6 themes: barriers to engagement, desired content, preferences for content delivery, preferences for interface, features, and aspects to avoid. Each theme had 2-7 subthemes that we relied upon when making design decisions for the prototype.

**Conclusions:**

Co-design workshops provided critical insights that informed the prototype development of a digital depression self-management tool tailored to AYA cancer survivors. Key takeaways that were integrated into prototype design include (1) using stories from other AYA cancer survivors to demonstrate concepts; (2) delivering content in brief lessons; and (3) using encouraging notifications, organizational tools, and reward systems to keep AYA cancer survivors engaged with the tool. Some of the themes identified in this study (eg, desired content and features) are consistent with known strategies for promoting user engagement and co-design work in other cancer survivors. However, this study extended previous research by identifying uniquely relevant strategies for tailoring to AYA cancer survivors, such as delivering content in brief sessions to overcome the time constraints AYA cancer survivors experience, providing opportunities for private expression, and maintaining an encouraging tone throughout the tool. These data were used to inform the prototype development of a digital depression self-management tool tailored to AYA cancer survivors.

## Introduction

### Depressive Symptoms Are Prevalent and Problematic in Adolescent and Young Adult Cancer Survivors

Annually, approximately 90,000 adolescents and young adults (AYAs) between the ages of 15 and 39 years are diagnosed with cancer in the United States, with over 2.1 million AYA cancer survivors living in the United States as of 2020. These numbers are expected to rise as the incidence of cancer in this age group increases [1,2]. Cancer survivors are up to 5 times more likely to experience depression than members of the general population [3]. Furthermore, a number of studies have demonstrated that younger cancer survivors are more likely to experience elevated depressive symptoms when compared with both older survivors and healthy age-matched peers [4-6]. Among AYA cancer survivors, estimates of the prevalence of depression range from 25% to 32% [7]. Elevated rates of depressive symptoms in AYA cancer survivors are likely due to disruptions or delays in the achievement of developmental milestones (eg, completion of education and becoming a parent) [8]. Depressive symptoms in AYA cancer survivors are problematic because depressive symptoms are associated with diminished health-related quality of life, increased health care use and costs, and elevated mortality [9].

### Psychosocial Interventions Are Efficacious in Treating Depressive Symptoms

The American Psychological Association’s clinical practice guidelines recommend cognitive behavioral therapy (CBT) for the treatment of depressive disorders in AYAs [10]. CBT focuses on helping patients recognize and change negative automatic thoughts (by cognitive restructuring) and maladaptive behaviors (by behavioral activation) that play a role in the development and maintenance of depressive symptoms [11]. Among adults, mindfulness-based cognitive therapy (MBCT) has also been shown to be efficacious. MBCT integrates elements of CBT with mindfulness-based stress reduction to improve one’s awareness of and relationship to unwanted thoughts, feelings, and bodily sensations [10,12]. More recently, there has been substantial interest in the use of positive psychology interventions to manage psychological symptoms in patients with severe and chronic illnesses. Systematic reviews and meta-analyses of trials of these interventions suggest they have small to moderate effects on depression and may be more effective than other depression interventions [13,14]. Thus, there are several topline evidence-based interventions (EBIs) for depression. Despite the elevated risk of depression in AYA cancer survivors and the existence of EBIs to address depression, it is unclear whether AYA cancer survivors can access such services. Indeed, the AYA Health Outcomes and Patient Experience study found that between 56% and 75% of AYA cancer survivors reported an unmet need for psychosocial support 12 months postdiagnosis [15].

### Digital Tools Can Increase Psychosocial Intervention Reach

Digital tools are a potential solution to overcoming barriers to AYA cancer survivors’ unmet needs for psychosocial support and a preferred mode of delivery for this population [16]. They allow interventions to be delivered remotely using smartphone technology that the vast majority of AYA cancer survivors already own, making them highly scalable [17]. Several studies have demonstrated the feasibility of digital tools for the self-management of physical symptoms among AYA cancer survivors [18]. Furthermore, research suggests psychosocial interventions for depression can be adapted for digital delivery with evidence for efficacy among healthy AYAs [19-25]. However, few existing digital interventions targeting depression have been evaluated in studies focused on AYA cancer survivors.

### Evidence for Digital Interventions Targeting Depression in AYA Cancer Survivors Has Been Mixed

In a 2015 meta-analysis, Richter et al [26] concluded there was limited evidence for the efficacy of existing interventions targeting psychosocial distress in AYA cancer survivors, including depressive symptoms. Since that time, Campo et al [27] published the outcomes of a mindful self-compassion videoconference intervention with 25 AYA cancer survivors and demonstrated improvements in depression following the intervention (Cohen *d*=0.99). Zhang et al [28] recently demonstrated the feasibility and preliminary efficacy of technology-assisted CBT for reducing depression in a pilot randomized controlled trial of 17 AYA cancer survivors (Hedges *g*=1.12). Although these findings are promising, others have been less so. For example, Sansom-Daily et al [29] found that an online group-based CBT intervention for AYA cancer survivors early in cancer survivorship was associated with a higher level of depressive symptoms compared with a control group (*P*=.04). As such, it remains unclear whether CBT, mindfulness, or other interventions will be most efficacious for improving depressive symptoms in this population. Furthermore, the interventions tested by both Campo et al [27] and Sansom-Daily et al [29] were delivered by interventionists via videoconference; while this strategy addresses some barriers to AYA cancer survivors’ participation in psychosocial interventions (eg, distance or travel-related expenses), they are not available on demand as AYA cancer survivors prefer [16].

### Content Tailoring and User-Centered Design Increase Efficacy and Engagement

While there is a growing body of available general mental health apps, these tools do not address the specific needs and concerns of AYA cancer survivors, such as negative thoughts about cancer and feelings of isolation from peers [30-32]. A significant consequence of this is low user engagement [33]. In the general population, only 1 out of 5 apps downloaded are used more than once [34]. The risk of nonuse of mobile health apps can be reduced with tailoring and effective design [35]. Tailoring involves developing individualized interventions based on key individual difference variables such as age and cancer history [36]. Data suggest that tailoring intervention content to the population of interest facilitates greater content relevance, which may result in more efficacious interventions and higher rates of long-term use [37]. User-centered design (UCD) processes allow for the integration of end-user input and feedback regarding features designed to increase user engagement [38]. For example, McCurdie et al [39] proposed a UCD process that consists of three iterative steps: (1) concept generation and ideation, (2) prototype design and system development, and (3) evaluation [39]. Following the final evaluation phase, the product is deployed. Users are at the center of this process, providing input at the concept generation and ideation step as well as the evaluation step. In this study, we aimed to engage AYA cancer survivors in the concept generation and ideation step of this process and design an initial prototype.

### Research Context and Guiding Strategy

The work presented in this paper forms part of a wider study informed by the multiphase optimization strategy (MOST) that aims to prepare and optimize a digital depression self-management tool for AYA cancer survivors. MOST is a comprehensive framework that can be used to optimize an intervention by systematically identifying the most promising components [40,41]. MOST includes three phases: (1) preparation, which lays the groundwork for optimization (eg, identification of a guiding conceptual model, choosing candidate components, formative work, and pilot-testing); (2) optimization, in which a trial (ie, a factorial experiment) is used to identify the components that meet the optimization criterion; and (3) evaluation, in which the effectiveness of the optimized intervention is confirmed. The work of this study was informed by the tasks to be completed during the preparation phase of MOST [42].

## Methods

### Overview

**Figure 1:**
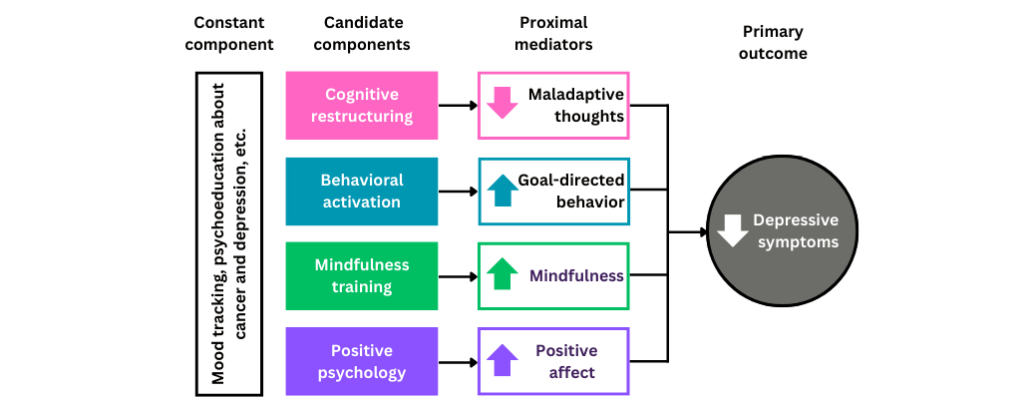
The guiding conceptual model for the preparation of a digital depression self-management tool for adolescent and young adult cancer survivors.

In alignment with guidance from MOST, we devised a theoretically and empirically supported model that informed our selection of candidate intervention components (Figure 1). We then engaged in an iterative UCD process to adapt these candidate intervention components for delivery through an existing self-guided digital tool and to address the unique needs and concerns of AYA cancer survivors to ensure user engagement. We began this process by conducting co-design workshops with AYA cancer survivors and oncology providers that serve this population to generate a concept for the adapted tool. At the same time, the end users of the digital tool will be AYA cancer survivors. Input from providers supplemented AYA cancer survivors’ suggestions with expertise regarding how this tool might overcome existing barriers to the management of depressive symptoms among AYA cancer survivors. Of note, the positive psychology component had already been modified for use with AYA cancer survivors and thus was not included in the co-design process [43]. Based on workshop findings, KM designed the prototype of the tool.

### Participants

Inclusion criteria for AYA cancer survivors were as follows: (1) diagnosed with cancer between the ages of 12 and 39 years; (2) currently between the ages of 15 and 39 years; (3) at least 1 month after completion of primary treatment; (4) fluent in spoken and written English; (5) have access to a smartphone with a data plan; and (6) have access to a computer with internet, a webcam, and a microphone that could be used to participate in a Webex (Cisco) videoconference. Notably, the National Cancer Institute (NCI) typically defines AYA cancer survivors as individuals diagnosed between the ages of 15 and 39 years and currently aged 15-39 years [1]. We chose to expand the age of diagnosis for adolescents as they are frequently diagnosed with hematologic malignancies, which require lengthy treatments such as stem cell transplants. Thus, by the time these patients reach posttreatment survivorship, they are considered emerging adults. Decreasing the age of diagnosis to 12 years for this subset of our sample ensured that adolescents currently aged 15-17 years were represented.

Inclusion criteria for oncology providers that serve AYAs were as follows: (1) licensed provider who treats AYA cancer survivors; (2) fluent in spoken and written English; (3) have access to a smartphone with a data plan; and (4) have access to a computer with internet, a webcam, and a microphone that could be used to participate in a Webex videoconference.

Potentially eligible participants were excluded if they reported a current diagnosis of a severe or persistent mental illness or reported severe suicidal ideation, including plan and intent. A safety protocol was in place to ensure appropriate care was provided if a potentially eligible participant reported suicidal ideation.

### Recruitment and Enrollment

AYA cancer survivors were recruited from an NCI-designated comprehensive cancer center in the Southeastern region of the United States. Potentially eligible AYA cancer survivors were identified through an electronic medical record review. First, an automated Epic (Epic Systems) report was used to identify patients in the correct age range who had been diagnosed with cancer. Study staff then conducted additional chart review to evaluate eligibility with regard to age at diagnosis, type of diagnosis, time since completion of primary treatment, and history of severe or persistent mental illness. Potentially eligible providers were identified based on their affiliation with an AYA oncology program within North Carolina.

Study staff contacted potentially eligible participants or (in the case of adolescents) their parents or guardians by email and (if screening was not completed and no response was received from the participant after 1 week) phone call. Interested individuals or (in the case of adolescents) their parents or guardians were directed to a website with more information about the study and asked to complete a REDCap (Research Electronic Data Capture; Vanderbilt University) [44] screening form to determine their eligibility. This screening form requested that prospective participants (or, in the case of adolescents, their parents on their behalf) provide their current age, age at cancer diagnosis, the type of cancer they had been diagnosed with, how long it had been since they completed cancer treatment, whether they had ever been diagnosed with a severe or persistent mental illness, whether they had access to a smartphone and computer, and their ability to speak and read English. Eligible and interested participants and (in the case of adolescents) their parents or guardians were automatically directed to the appropriate assent and consent forms (see the *Ethical Considerations* subsection for a detailed explanation of the assent and consent process).

Upon provision of assent and consent, AYA cancer survivors and providers were asked to provide their demographic information and availability for participation in study procedures. Co-design workshop participants were scheduled in groups of at least 3 participants.

### Co-Design Workshops

#### Overview

KMM conducted 4 online co-design workshops with AYA cancer survivors and 1 co-design workshop with providers that serve this population using Webex [45]. AYA cancer survivors and providers completed workshop activities from their homes or offices. No one else was present during workshops other than the participants and the researcher. KMM is a female individual with a PhD in Clinical Psychology and, at the time of the interview, was an instructor at Wake Forest University School of Medicine. KMM has extensive training in qualitative data collection methods and completed a certificate in UCD. She did not have a relationship with AYA cancer survivor participants before the study commencement but had reviewed their demographic and clinical characteristics. Regarding providers, KMM had previously interacted with some of the participants in professional settings but did not have any formal working relationship with them. As the principal investigator of this study, KMM’s primary interest in the research topic was informing the development of a digital tool to help AYA cancer survivors better manage symptoms of depression, and this was communicated to all participants. Providers and AYA cancer survivors attended separate workshops. Each workshop lasted approximately 1 hour. Workshops were audio and video recorded and autotranscribed.

#### Workshop Activities

**Figure 2:**
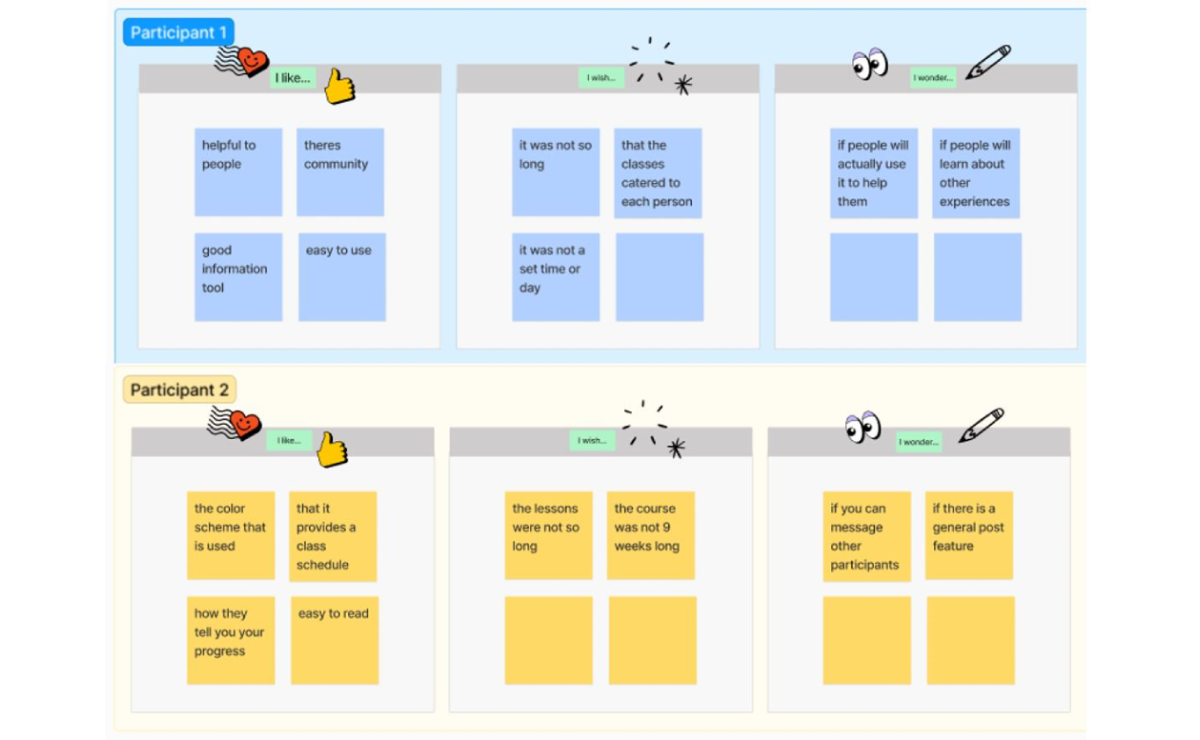
Sample responses to the “I like, I wish, I wonder” activity from the adolescent and young adult cancer survivor workshop.

**Figure 3:**
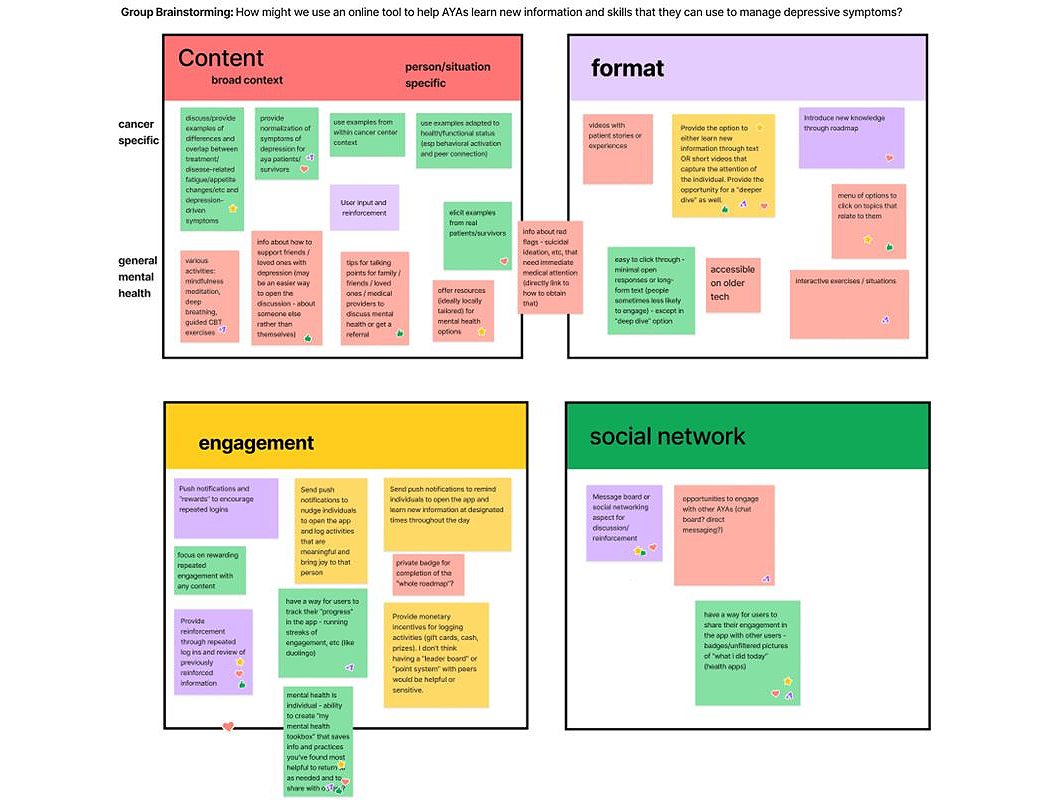
Sample responses to the group brainstorming activity from the provider workshop. AYA: adolescent and young adult.

To begin each workshop, participants were provided with information about the goal of the project, namely, to develop a tool that helps AYA cancer survivors manage symptoms of depression. In addition, participants were introduced to EBIs for depression (eg, behavioral activation and mindfulness training) that we aimed to adapt and tailor for digital delivery to AYA cancer survivors. After providing some general guidelines for brainstorming and an introduction to Figma (Figma, Inc) [46] (a digital whiteboard tool), participants were asked to provide feedback on an existing digital mindfulness course (Wakeful) using an “I like, I wish, I wonder” prompts within Figma (Figure 2). In this context, wakefulness was presented to participants as an example to spur ideas. Then, participants were asked “How might we...” questions focused on brainstorming ideas for how the digital tool might work (Figure 3). Participants first brainstormed responses to each question independently and then worked as a group to categorize and expand on their ideas. Slides presented during workshops are included as Multimedia Appendix 1 (AYA cancer survivor workshops) and Multimedia Appendix 2 (provider workshop).

### Data Processing and Analysis

Field notes were made by the researcher following each workshop. Co-design workshops were autotranscribed using Webex software. Transcripts of co-design workshops were cleaned by study team members with additional context provided by workshop products (ie, sticky notes). Reference was made to the original audio and video recordings as needed for clarification. Transcripts were not returned to participants for correction, given the availability of audio and video recordings.

RG, with oversight from KMM, conducted a thematic analysis of workshop transcripts guided by the methodological framework by Braun and Clarke [47]; the analysis was characterized by an inductive approach, where themes were directly derived from the transcripts rather than predefined before the analysis. The analysis began with a familiarization phase of understanding the content, which provided initial insights into overarching themes. Following familiarization, the coding phase included identifying and assigning a descriptive code that succinctly encapsulated the content. These codes systematically organized the data and identified patterns that later informed the identification of themes. The thematic analysis provided a comprehensive understanding of the data, highlighting the participant interactions within the co-design workshops. Participants did not provide feedback on the findings; rather, findings were used to develop a prototype that was then presented to additional participants to gather feedback.

### Ethical Considerations

All study procedures were reviewed and approved by the Wake Forest University Health Sciences Institutional Review Board (IRB00075887). This included an informed consent and assent process, which primarily occurred through REDCap. Prospective participants and (in the case of adolescents) their parents were provided with a summary of the study, information about why the study was being conducted, the procedures involved in participating in the study, the duration of the study, the designation of the study as no more than minimal risk, a description of the minimal risks inherent in participating in the study (eg, potential for breach of confidentiality or psychological discomfort), the benefits to participating in the study (eg, helping others in the future), alternatives to participating in the study, compensation for participating in the study, and contact information for the principal investigator and Institutional Review Board.

In the case of young adults, eligible participants were asked to review this information about the study and required to give their consent to participate before providing their demographic and clinical information and participating in the workshop. In the case of adolescents younger than the age of 18 years, study staff initially contacted the parent of the patient. Parents were asked to review information about the study and provide their permission for the adolescent to participate. Upon provision of parental permission, study staff contacted the adolescent directly. Adolescents were provided with age-appropriate information about the study through REDCap and were required to provide their assent to participate before providing their demographic and clinical information and participating in the workshop.

All remote data collection occurred through Health Insurance Portability and Accountability Act (HIPAA)–compliant platforms, and all identifiable data were stored on secure, password-protected servers (eg, REDCap and institutional servers) only accessible by authorized study staff. Recordings of workshops were transcribed and deidentified for analysis. All information identifying participants will be deleted upon publication of the primary study outcomes.

Participants were compensated US $40 for participating in a workshop. Compensation was provided in the form of a digital gift card that was emailed to the participant following the workshop.

## Results

### Participants

In total, 4 providers and 8 AYA cancer survivors participated in 5 co-design workshops, consistent with the recommended number of focus group discussions needed to achieve saturation [48]. There were 2 additional AYA cancer survivors who expressed interest in the study but did not ultimately participate in a workshop due to scheduling conflicts. The provider workshop had 4 participants, while each of the AYA cancer survivors’ workshops had 2 participants. All workshops lasted approximately 1 hour.

The majority of providers identified as non-Hispanic White (4/4, 100%) and heterosexual (3/4, 75%). Disciplines represented by providers included medical oncology and social work. AYA cancer survivors ranged in age from 15 to 37 years (mean 23.5, SD 8.85 years). The majority of AYA cancer survivors identified as non-Hispanic White (6/8, 75%), female (6/8, 75%), and heterosexual (7/8, 87.5%). Each AYA cancer survivor had been diagnosed with cancer between the ages of 12 and 36 years. All AYA cancer survivors had completed treatment between 1 month and 5 years before participating in the workshop.

### Thematic Analysis

Thematic analysis revealed 6 overarching themes: barriers to engagement, preferences for content delivery, desired content, preferences for interface, features, and aspects to avoid. Each of these themes and their subthemes are described in detail below, with exemplar quotes. Of note, some quotes were spoken by participants, and this was pulled from the transcript; other quotes were never spoken by participants but instead typed out on the Figma board as part of engaging with the digital workshop materials. As such, we have denoted whether quotes are from the “Transcript” or “Workshop Materials,” respectively.

### Theme 1: Barriers to Engagement

This theme represents the challenges participants might face that hinder their ability to engage effectively with the tool. These barriers are crucial in understanding the obstacles that must be addressed to enhance participation and involvement. The 2 primary barriers were time constraints and lack of usability.

#### Subtheme 1.1: Time Constraints

Overall, the participants emphasized challenges with time constraints due to balancing work, school, and family responsibilities. They highlighted that these time constraints often lead to difficulties in consistently engaging in activities to support their needs, as highlighted by the quote below.

Yeah, probably, um, lesser time periods, maybe spread them out a little bit more. Um, For the busy momma’s out there, 'cause I tell ya when you work 80+ hours a week and go to school full time and have a family.

24-Year-old leukemia survivor, transcript

#### Subtheme 1.2: Lack of Usability

A significant barrier noted by both groups of participants, particularly older AYA cancer survivor users and providers, is a lack of usability. This includes challenges related to outdated technology and user interfaces that may not be intuitive for this demographic. Addressing these usability issues is essential to enhance the overall user experience and to ensure the tool is accessible.

Okay, I’m like the older AYA cancer survivors, and I’m struggling with my brain. It’s trying to, like, learn the new technology before it can think...so, I almost feel like, I can’t think.

37-Year-old breast cancer survivor, transcript

There would be attention paid, and that the design would be such that it would be accessible on, like, multiple, even maybe older operating systems. Because especially if these people are dealing with the financial toxicity of cancer treatment. Their tech may be a couple of versions back. So, I guess paying attention to accessibility and ease of use.

Provider 3, medical oncologist, transcript

### Theme 2: Desired Content

This theme represents the types of content that participants find most valuable and engaging. Understanding these preferences helps ensure that the content is relevant, engaging, and aligned with AYA cancer survivors’ interests and needs. We identified 4 specific types of content: information about mental health, resources, AYA cancer survivors’ stories, and cancer-specific content.

#### Subtheme 2.1: User Guidance

Participants expressed a desire for the tool to include a comprehensive guide for the tool. They indicated that having a structured guide would help them navigate the tool more effectively, understand its features, and maximize its functionalities.

I think, yeah, there could be like a guide like, when you first open the app or like, uh, not really a tutorial, but like sort of have it go through the app by its features and stuff. I think that would be pretty helpful.

15-Year-old gastrointestinal stromal tumor (GIST) survivor, transcript

#### Subtheme 2.2: Information About Mental Health

Participants and providers expressed a strong desire for mental health information to be integrated throughout the tool. They emphasized the importance of incorporating relevant mental health content, such as information about depression, to address the specific needs and concerns of AYA cancer survivors.

How depression can start, what depression can lead to, and what I need to do if I am depressed.

19-Year-old brain tumor survivor, workshop materials

#### Subtheme 2.3: Resources

Participants and providers expressed a strong desire for the tool to include a variety of resources that address different aspects of the AYA cancer survivors’ cancer experiences. This includes a desire to have information about how to cope with mental health difficulties; rediscovering enjoyable activities after cancer; and external resources, particularly for emergent mental health concerns. Incorporating these elements will ensure that the tool offers comprehensive support and valuable information tailored to the needs and interests of AYA cancer survivors.

Information about immediate medical attention for suicidal ideation.

Provider 1, medical oncologist, workshop materials

Have helpful tools outside of this for people to manage their depression on a daily basis.

20-Year-old thyroid cancer survivor, workshop materials

This is actually a tool to help me figure out who am I now after going through this...Like, okay, I tried soccer again and on a scale of 1 to 10, I enjoyed, you know, like. More or less some sort of, I think, it would feel more. Um, I don't know what the word is like, less like, you feel a little bit, it would feel a little more empowering, I think, and a little bit less like, there's something else cancer has taken from you.

37-Year-old breast cancer survivor, transcript

Mental health is individual—ability to create “my mental health toolbox” that saves info and practice you’ve found most helpful to return to as needed and to share with others.

Provider 3, medical oncologist, workshop materials

#### Subtheme 2.4: AYA Cancer Survivors’ Stories

Both participants and providers highlighted the importance of including stories from AYA cancer survivors within the tool. They emphasized that featuring personal experiences from peers can provide valuable insights, foster a sense of connection, and offer relatable examples that resonate with the AYA cancer survivors’ community.

I think you could give, like, ask people if they would volunteer to give like their stories you could have certain like videos or articles from them that showed how they got through it sort of like a motivation.

15-Year-old GIST survivor, transcript

Include videos with patient stories or experiences.

Provider 1, medical oncologist, workshop materials

#### Subtheme 2.5: Cancer-Specific Content

Providers noted that incorporating cancer-specific content could be highly beneficial. This specialized content can enhance the tool’s relevance and effectiveness by offering targeted guidance and support for those affected by cancer.

Discuss/provide examples of differences and overlap between treatment/disease-related symptoms and depression-driven symptoms.

Provider 2, social worker, workshop materials

### Theme 3: Preferences for Content Delivery

This theme represents how participants prefer to receive content, which is essential for tailoring to meet the needs of users who are AYA cancer survivors. Understanding these preferences helps ensure that content is delivered in a way that aligns with participants’ schedules and learning styles. We identified 3 important aspects of content delivery: intervention duration, session duration, and learning modalities.

#### Subtheme 3.1: Intervention Duration

Participants preferred shorter intervention durations, reflecting the multiple responsibilities that AYA cancer survivors juggle. They indicated a reduced timeframe would better accommodate their busy schedules and enhance their ability to engage consistently.

I just wish it wasn’t as long as a nine-week course because, like, with me, I’m trying to go to school and, like, work at the same time. So, like it’d be nice if like, this was something that I can do, like, after school, or like between school and work. So that way it wouldn’t take so long.

20-Year-old thyroid cancer survivor, transcript

#### Subtheme 3.2: Session Duration

Participants expressed a desire for shorter session durations to better accommodate AYA cancer survivor users’ busy schedules. They highlighted that reduced session lengths would make it easier for them to fit the sessions into their diverse and demanding routines.

Uh, probably just to make it shorter, I don’t want to like be on here for a long time.

19-Year-old brain tumor survivor, transcript

I guess if I could change one thing about it, it would be lessening the class time.

24-Year-old leukemia survivor, transcript

#### Subtheme 3.3: Learning Modalities

Both participants and providers expressed a desire for varied learning and interaction modalities. They emphasized the need for diverse content delivery methods to cater to different learning styles and preferences.

I liked how like there were scripts for the videos so, in case someone doesn't want to, like, watch a video. They can just read through it.

15-Year-old GIST survivor, transcript

Provide the option to either learn new information through text or short videos.

38-Year-old breast cancer survivor, workshop materials

### Theme 4: Preferences for Interface

This theme represents participants’ preferences regarding the design and functionality of the tool’s interface. Understanding these preferences helps ensure that the interface is intuitive, user-friendly, and visually engaging. We identified 2 preferences for interface design: ease of use and color scheme.

#### Subtheme 4.1: Ease of Use

Both participants and providers emphasized the importance of a user-friendly interface, highlighting the need for intuitive navigation and accessibility to ensure AYA cancer survivor users can interact with the tool efficiently and comfortably. Participants prefer that the tool resumes from where they left off, allowing for a seamless and uninterrupted user experience.

I like that it [a sample interface] is very easy to read. It looks like it looks very user friendly as well.

24-Year-old leukemia survivor, transcript

It is important that the tool is easy to click through—minimal open responses.

Provider 3, medical oncologist, workshop materials

#### Subtheme 4.2: Color Scheme

Participants had varied opinions on the tool prototype’s color scheme. Some expressed enjoyment of the current design, appreciating its visual appeal. However, others felt that the color scheme was more suited to a younger audience, suggesting a need for a more age-appropriate or versatile design to better align with the preferences of all AYA cancer survivor users.

Personally, I like it [a sample interface] a lot. Like, I like, how all the colors kind of like I don’t know, I don’t know how to put it. Um, blend well, I guess.

15-Year-old thyroid cancer survivor, transcript

I feel like I like the colors too, but I think it [a sample interface] sort of it's more of a younger audience all like the bright colors and stuff. I think if it's designed for all ages, I think there should be, like, uh, three colors that you use, and not just like all over the color spectrum.

15-Year-old GIST survivor, transcript

### Theme 5: Features

This theme represents the essential functionalities and characteristics that participants consider important for an effective tool. Identifying and integrating these features ensures that the tool meets the diverse needs of AYA cancer survivor users, making it more engaging, user-friendly, and supportive. We identified 7 functionalities participants indicated would be important to include in the tool: daily mood tracking, encouraging notifications, organizational tools, reward system, interactive exercises, connection, and private expression.

#### Subtheme 5.1: Daily Mood Tracking

Participants preferred daily mood tracking instead of hourly updates. They indicated that daily tracking is more manageable, less intrusive, and aligns better with their routines. Participants thought that such “check-ins” would be helpful as they provide valuable opportunities for self-reflection.

15-Year-old GIST survivor (transcript): I think, yeah, I like how it would just be like, for the whole day, instead of each hour.

Like um, mostly I'm thinking of a message that would ask how were doing.

KMM: Yeah, yeah, so kind of like a check-in.

21-Year-old large B cell lymphoma survivor (transcript): Yes, exactly.

15-Year-old GIST survivor (transcript): Yeah, yeah, it [mood tracking by the hour] makes some might feel overwhelmed.

#### Subtheme 5.2: Encouraging Notifications

Participants and providers noted the effectiveness of using notifications within the tool to promote user engagement. They highlighted that encouraging notifications may facilitate consistent interaction with the tool and aid in maintaining a positive outlook.

Yeah, I think just to get a reminder, like some days you might forget to add like your thoughts or whatever, I think just having some sort of notifications, maybe just like, once a day.

15-Year-old GIST survivor, transcript

Send push notifications to nudge individuals to open the app and log activities that are meaningful and bring joy to that person.

Provider 1, medical oncologist, workshop materials

#### Subtheme 5.3: Organizational Tools

Both participants and providers valued the integration of organizational elements into the tool, such as a to-do list, distinct weekly sessions, and goal or progress tracking. These features provide users with a structured framework, helping them manage tasks, stay organized, and track their progress. In addition, participants enjoyed the session schedule and progress monitoring features of an exemplar interface.

Include a To-do list kind of option.

15-Year-old thyroid cancer survivor, workshop materials

Introduce new knowledge through roadmap.

Provider 3, medical oncologist, workshop materials

#### Subtheme 5.4: Reward System

Both participants and providers recommended incorporating reward systems to enhance user engagement. These reward systems can provide positive reinforcement, recognize achievements, and encourage consistent participation.

I also like when you have sort of point or reward system. So, you can, I guess it gives you more motivation to what your thoughts and ideas on there.

15-Year-old GIST survivor, transcript

Provide monetary incentives for logging activities (gift cards, cash, prizes).

Provider 1, medical oncologist, workshop materials

#### Subtheme 5.5: Interactive Exercises

Providers recommended incorporating interactive elements into the exercises and situations within the tool.

Include interactive exercises/situations.

Provider 1, medical oncologist, workshop materials

#### Subtheme 5.6: Connection

Participants and providers wanted users to have the option to connect with other AYA cancer survivors through the tool as was shown in an exemplar interface. They suggested that this feature could facilitate meaningful connections and encourage mutual support among users.

I like how you can chat with the other people to experience the app together and talk with each other about your experience.

20-Year-old thyroid cancer survivor, transcript

I like that you have other people to chat with and can experience the course together.

38-Year-old breast cancer survivor, transcript

Message board or social networking aspect for discussion/reinforcement.

Provider 2, social worker, workshop materials

#### Subtheme 5.7: Private Expression

Participants expressed a desire for a feature within the tool that ensures the confidentiality and security of their emotions and thoughts. They emphasized the importance of having a safe space to express themselves without concerns about privacy.

There’s some type of place where you don't have to worry at all about who’s gonna see it or what’s gonna see it, and you can just put it, and it’s either completely just for you or you can give, like, only up to 3 people access, like, some sort of like the vault of all of my horrible feelings can go to this place. And I can just sort of dump in there, even if it's like, even if it is just for the person and just for them to see later, I think there's some value in doing that.

38-Year-old breast cancer survivor, transcript

### Theme 6: Aspects to Avoid

Both participants and providers identified undesirable or problematic strategies for engagement. Addressing these concerns is crucial to ensure that the tool is user-friendly, engaging, and aligned with the needs of AYA cancer survivor users. We identified competition and negative tone as challenges to engagement.

#### Subtheme 6.1: Competition

This theme highlights the importance of avoiding features that may foster competition between participants. Providers suggested that ensuring the tool promotes a positive, non-competitive experience is essential for maintaining user engagement and fostering a sense of community.

Avoid having a “leaderboard” or “point system” with peers.

Provider 1, medical oncologist, workshop materials

#### Subtheme 6.2: Negative Tone

Participants emphasized the need to avoid negative or potentially discouraging content. It is important to ensure that all content is supportive, uplifting, and encouraging.

People who have a lot of self-hate towards themselves that might wanna want to put negative responses towards themselves that could possibly make them feel worse.

21-Year-old survivor of multiple cancers, transcript

### The Prototype Depression Self-Management Tool

Based on feedback and recommendations from workshop participants, the initial design of the tool was determined by the study team (see Table 1 for a summary of how each theme and subtheme informed design decisions). There were a few conflicts between the providers’ and AYA cancer survivors’ suggestions; however, in the case of conflicting suggestions, AYA cancer survivors’ preferences were prioritized as they are the target end users of the digital tool.

**Figure 4:**
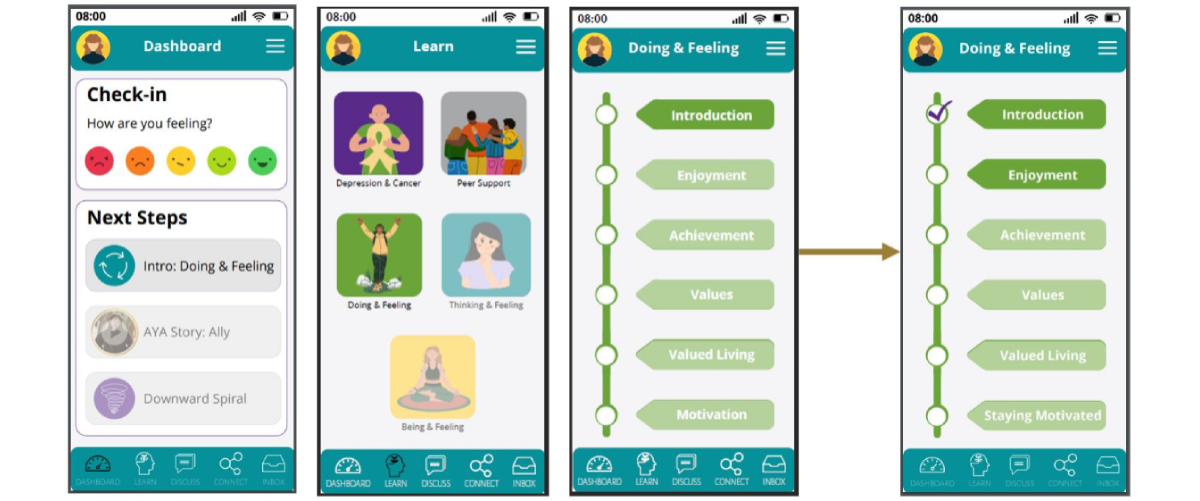
Dashboard and learning modules.

**Figure 5:**
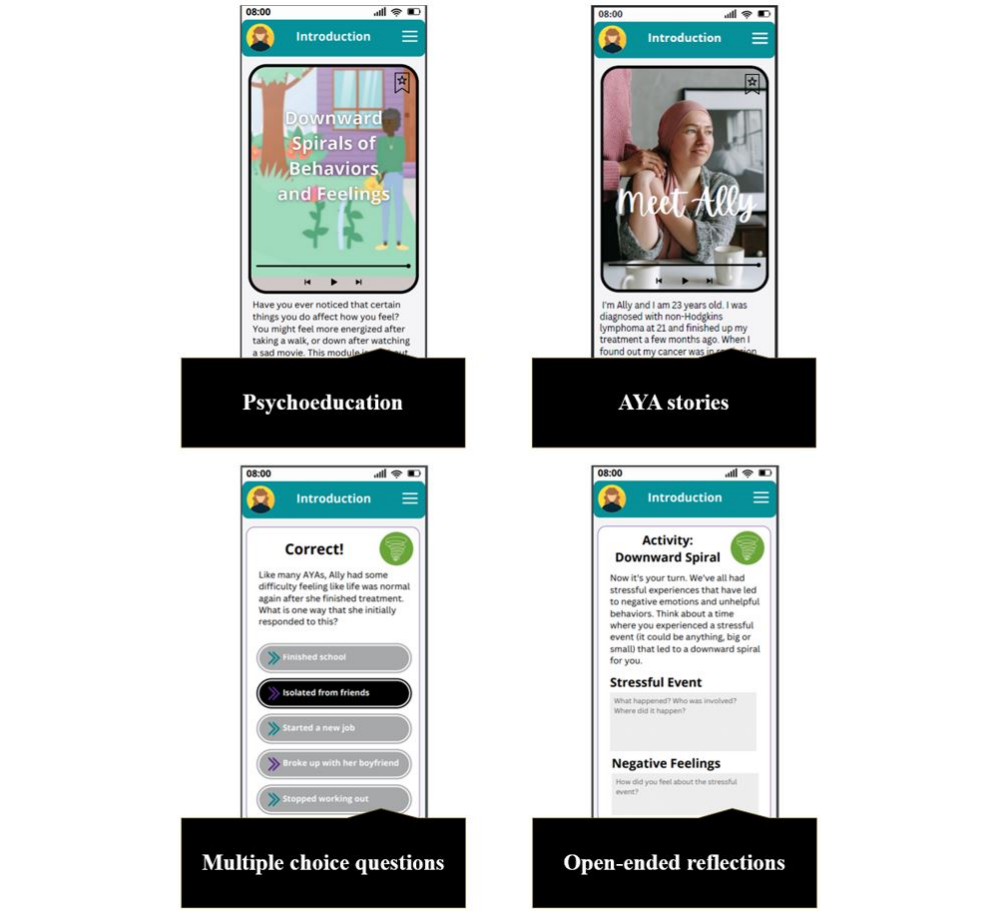
Example lesson structure and design. AYA: adolescent and young adult.

KM used Canva (Canva Inc) [49] to design a prototype of a depression self-management tool tailored to the needs and preferences of AYA cancer survivors. The tool was designed with the aim of providing mood tracking, psychoeducation about depression and cancer, and 4 adapted evidence-based interventions (aEBIs) for depression to this population via a web-based, app-optimized smartphone delivery (Figure 4). Each aEBI was designed to be housed in its own module, which would consist of a series of brief lessons. Each lesson would include educational content regarding the lesson topic (presented via video and transcript), a story of an AYA cancer survivor that illustrated the lesson topic, app questions that asked the user to apply the educational information to the AYA cancer survivors’ story, an activity in which they reflected on what they learned related to their experience, and a practice activity that asked users to apply what they learned in their day-to-day life (Figure 5). The tool also featured a discussion board section that would allow users to connect with one another about the content presented via the tool.

**Table 1:** Summary of how each theme and subtheme informed design decisions.

Theme and subtheme			Design decision
**Barriers to engagement**			
	Time constraints	Each aEBI^a^ will be delivered via a series of brief, on-demand lessons that users can complete when it is convenient for them.	
	Lack of usability	A web-based application with a responsive design that has established usability among AYA^b^ cancer survivors will be adapted for content delivery.	
**Desired content**			
	User guidance	During research studies, participants will complete an orientation session that includes instructions on how to use the digital tool. Once the tool has been optimized, a user guide will be developed and provided to users.	
	Information about mental health	Relevant mental health information will be included in the constant component so that it is provided to all participants. External resources for emergent mental health concerns will be provided to users via the constant component.	
	Resources	Each aEBI will include relevant practice activities to help users apply content in their daily lives. Future research will inform how to best integrate these practice activities into the digital tool.	
	AYA cancer survivors stories	The tool will use AYA cancer survivors' stories to illustrate educational content.	
	Cancer-specific content	The constant component and aEBIs will include information that is tailored to AYA cancer survivors.	
**Preferences for content delivery**			
	Intervention duration	Content will be delivered over a duration shorter than 9 weeks. Additional research will inform intervention duration.	
	Session duration	The constant component and each aEBI will be delivered via a series of brief lessons. Additional research will inform session duration.	
	Learning modalities	Educational content will be presented via multiple modalities, such as a brief video with a transcript provided. Additional research will inform what content should be presented via each modality.	
**Preferences for interface**			
	Ease of use	A web-based application with a responsive design that has established usability among AYA cancer survivors will be adapted for content delivery.	
	Color scheme	The color scheme will be updated to reflect the age/maturity level of the target user population.	
**Features**			
	Daily mood tracking	Users will be asked to track their mood daily rather than hourly.	
	Encouraging notifications	Users will receive encouraging text message reminders to engage with the tool. Additional research will inform the frequency of these messages.	
	Organizational tools	The tool will display users’ progress toward completion of content and provide them with clear next steps.	
	Reward system	Additional research will be conducted to determine how to reward users for engagement in a way that is feasible and scalable.	
	Interactive exercise	Users will be asked to apply the information they learn to the stories of other AYA cancer survivors as well as their own experiences. Future research will explore how users prefer to input such responses in the tool.	
	Connection	The tool will include discussion boards that allow users to connect with other AYA cancer survivors.	
	Private expression	Users will be able to choose whether their responses to reflection questions are shared with other users.	
**Aspects to avoid**			
	Competition	Users will not be able to see the progress of other users.	
	Negative tone	A positive and encouraging tone will be used throughout the tool.	

^a^aEBI: adapted evidence-based intervention.

^b^AYA: adolescent and young adult.

## Discussion

### Principal Findings

AYA cancer survivors often experience depressive symptoms that go unaddressed. Digital mental health tools are a promising strategy to ensure that patients have the skills and tools necessary to manage symptoms of depression. This study described the initial co-design of a depression self-management intervention to ensure that the final product is tailored to the needs and preferences of AYA cancer survivors, fulfilling the concept generation and ideation step of the UCD process set forth by McCurdie et al [39] and contributing to the tasks of the preparation phase of the MOST framework [42]. Feedback from AYA cancer survivors during the co-design workshops provided critical insights into ways in which a digital tool could be tailored to engage this population in evidence-based strategies for depression self-management. Specifically, users emphasized the importance of overcoming barriers to engagement, such as time constraints; provided suggestions for content to be included in the tool and how it should be delivered; and recommended features to enhance engagement with the tool. Using these findings, we designed a midfidelity prototype of a digital depression self-management tool tailored to the needs and preferences of AYA cancer survivors.

### Comparison With Previous Work

A primary goal of the UCD process in this project was to ensure the tool we develop is engaging for AYA cancer survivors. As such, many of the themes identified in this study are consistent with previously identified strategies for promoting user engagement. For example, a systematic review of technology-supported strategies in digital mental health interventions identified four categories of engagement strategies used in existing tools: (1) reminders to use the tool, (2) tailored information, (3) peer support, and (4) coaching techniques [50]. In our study, the first 3 of these categories were emphasized by participants as important to include in the digital tool we are designing.

The themes identified in this study also broadly align with findings from co-design work in other groups of cancer survivors. For example, Adler et al [51] engaged cancer survivors with physical disabilities in a co-design process of a self-management tool. Their team identified themes of “engaging” and “socialization” that have significant overlap with our “features” theme. Participants in both studies suggested the use of participative features, progress tracking, and opportunities to connect with others who have had similar experiences as strategies to help users engage with a self-management tool. Similarly, in a co-design study focused on a tool to help cancer survivors engage in healthy behaviors, Leske et al [52] identified relatability and relevance as a theme, which has considerable overlap with the AYA stories and cancer-specific content subthemes of our desired content theme [52]. In both studies, it became evident that providing cancer-specific information and stories from peers would enhance the relevance of the tool for the target population.

Finally, this study extended previous research by engaging a novel population of AYA cancer survivors and their providers in the co-design process. This allowed us to elucidate strategies for tailoring that are uniquely relevant to this population, such as the importance of ensuring that the content is delivered in brief sessions to overcome the time constraints AYA cancer survivors experience, providing opportunities for private expression of their thoughts and feelings, and using a positive and encouraging tone throughout the tool.

### Limitations

Our findings are limited by the size and characteristics of our research sample and the use of web-based rather than in-person activities. Our total sample size of 12 individuals across 5 workshops is consistent with recommendations for reaching saturation and sample sizes in design research with similar populations [48,53]. However, this small group of participants inherently limits the generalizability of this study. In addition, despite efforts to schedule as many AYA cancer survivors for the same workshop as possible, difficulties in scheduling resulted in groups of 2. These group brainstorming sessions would have benefited from larger groups with more people to generate ideas. Furthermore, despite efforts to recruit a diverse group of AYA cancer survivors and providers, we did not achieve a representative research sample with regard to race, ethnicity, gender, and sexual orientation. However, our AYA cancer survivors sample was representative of the vast majority of the AYA cancer survivors’ age range and common cancer diagnoses. Finally, the activities we were able to engage AYA cancer survivors in were limited to the capabilities of existing digital tools; we were not able to use the materials typically relied upon during in-person, co-design sessions, such as markers, Post-it notes, and posters. At times, we also encountered technical difficulties with the tools that limited user engagement in co-design activities. For example, one user was not able to load Figma on her device during the workshop, so she was limited to adding her insights verbally or through the chat feature of Webex. However, the use of online tools for co-design ultimately provided invaluable flexibility in terms of location and scheduling to ensure the engagement of busy AYA cancer survivors in the process.

### Conclusions

This co-design study improved our understanding of the needs and preferences of AYA cancer survivors for a digital tool that may help them better manage symptoms of depression. This process allowed us to develop a shared vision of what such a digital tool would look like and how it would work. As such, our findings informed the development of a prototype of the tool. In addition, the findings from this work provide insights into the preferences of AYA cancer survivors for digital mental health tools more broadly. For example, AYA cancer survivors broadly agreed on the need for brief intervention models. However, it remains to be seen whether delivering interventions in a brief manner negatively impacts efficacy. As such, findings from this study are likely informative for future research aimed at designing engaging digital interventions for AYA cancer survivors. The next phase of this study is to have AYA cancer survivors evaluate the prototype through semistructured individual interviews to inform the design of a working prototype. This formative work will continue to contribute to the tasks set forth by the preparation phase of MOST, as will a subsequent pilot study of the tool.

## Appendix

Multimedia Appendix 1 Adolescent and young adult cancer survivor workshop slides.

Multimedia Appendix 2 Provider workshop slides.

## Abbreviations

aEBI: adapted evidence-based intervention
AYA: adolescent and young adult
CBT: cognitive behavioral therapy
EBI: evidence-based intervention
GIST: gastrointestinal stromal tumor
HIPAA: Health Insurance Portability and Accountability Act
MBCT: mindfulness-based cognitive therapy
MOST: multiphase optimization strategy
NCI: National Cancer Institute
REDCap: Research Electronic Data Capture
UCD: user-centered design

## Data Availability

The datasets generated or analyzed during this study are available in the Open Science Framework repository [54].
