# Supplementary material for: Co-Design of a Depression Self-Management Tool for Adolescent and Young Adult Cancer Survivors: User-Centered Design Approach
Source: JMIR Form Res. 2025 Mar 24;9:e67175. doi: 10.2196/67175 (PMC11976180; doi:10.2196/67175)
Supplement: Multimedia Appendix 2 [file formative_v9i1e67175_app2.pptx]

## Slide 1
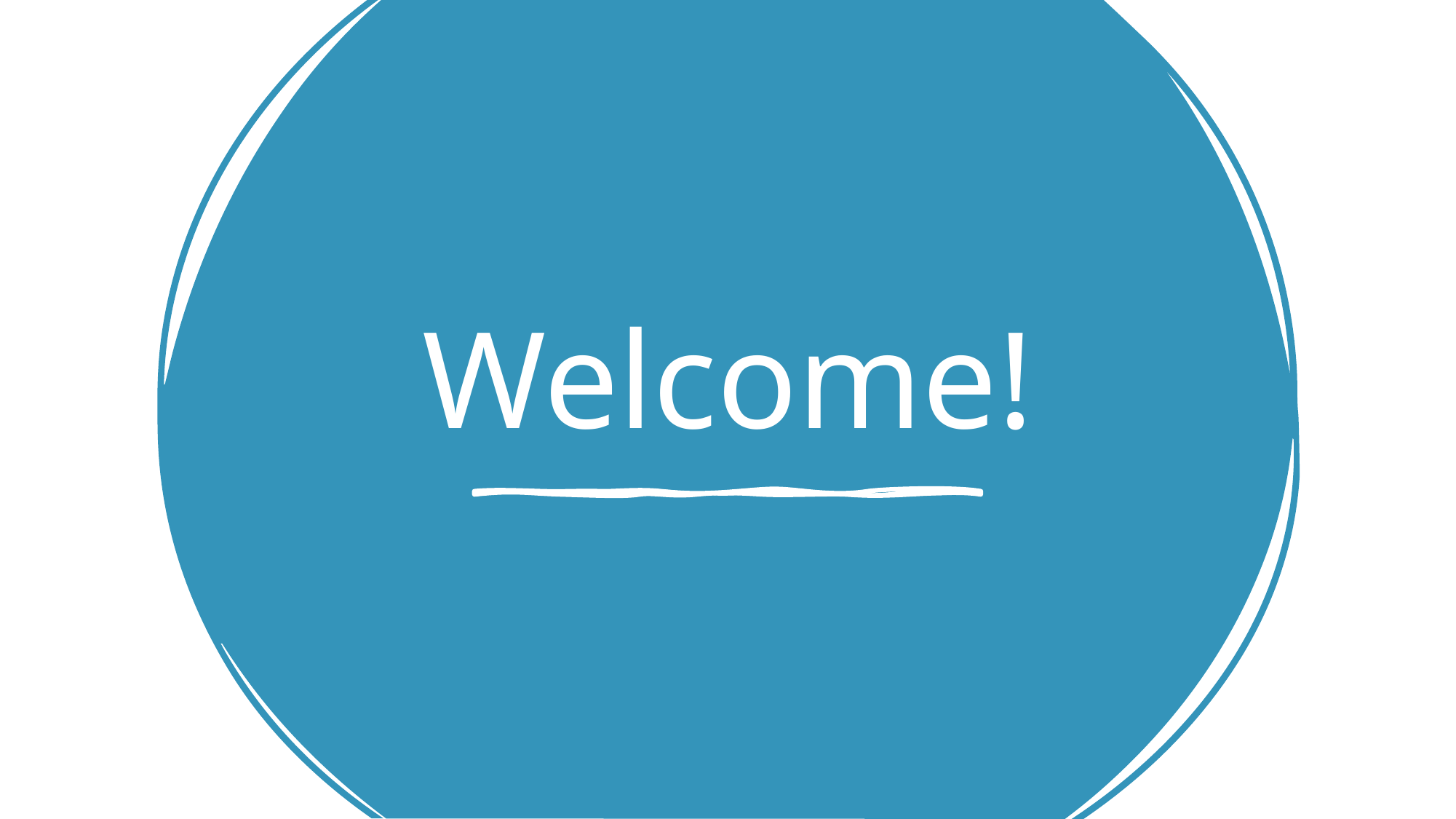

# Welcome!

## Slide 2
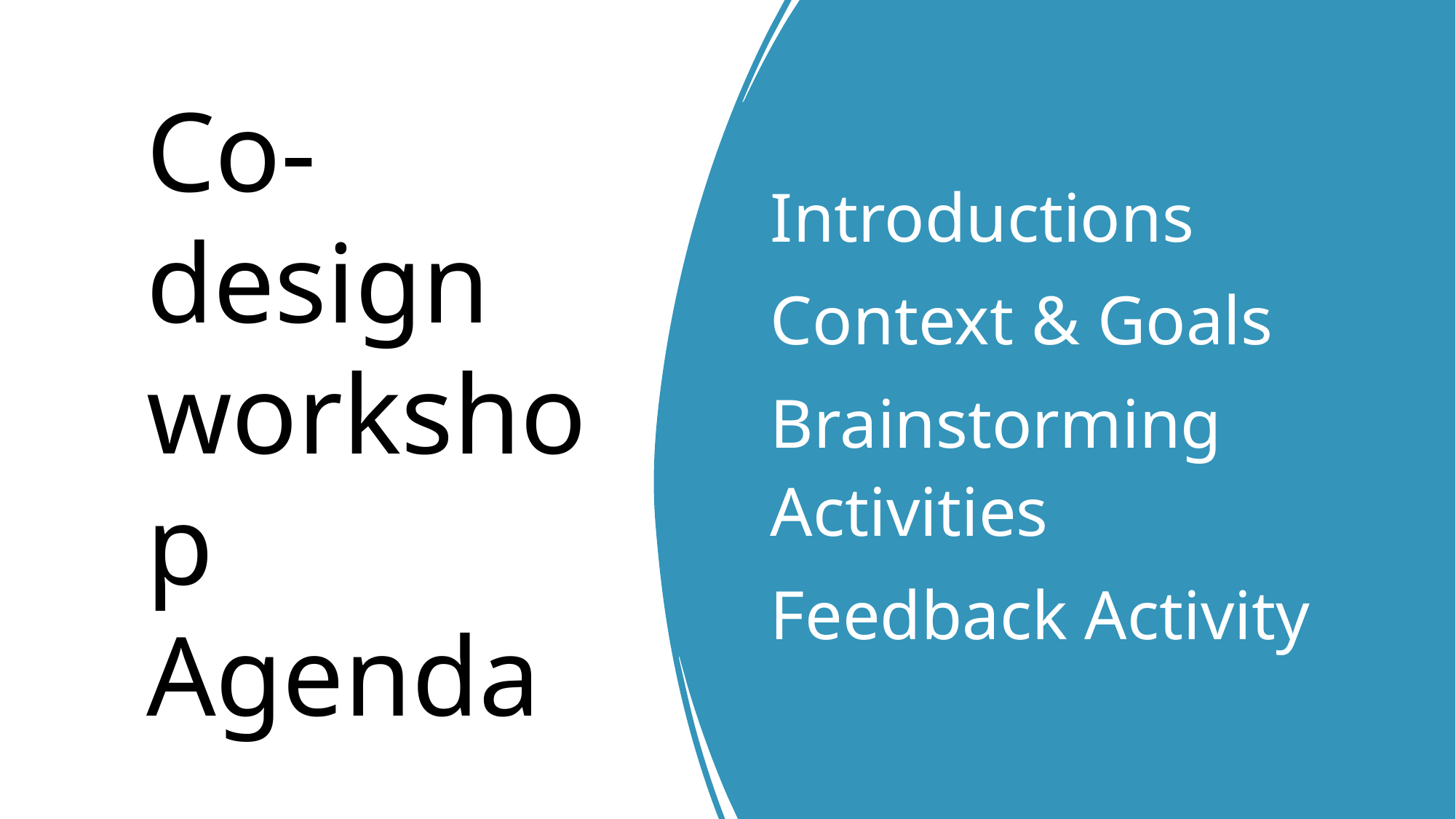

# Co-design workshop Agenda
Introductions
Context & Goals
Brainstorming Activities
Feedback Activity

## Slide 3
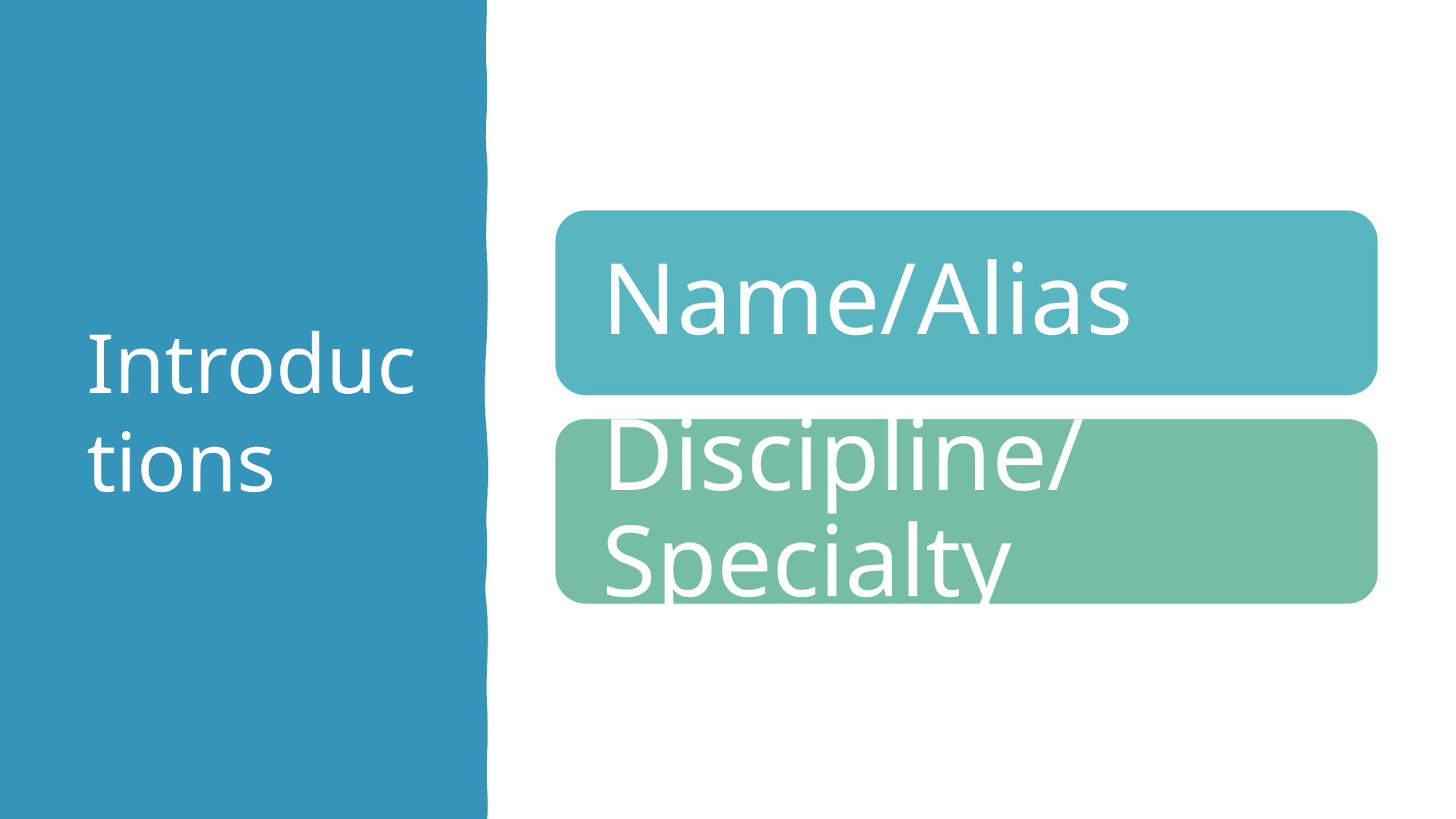

# Introductions

## Slide 4
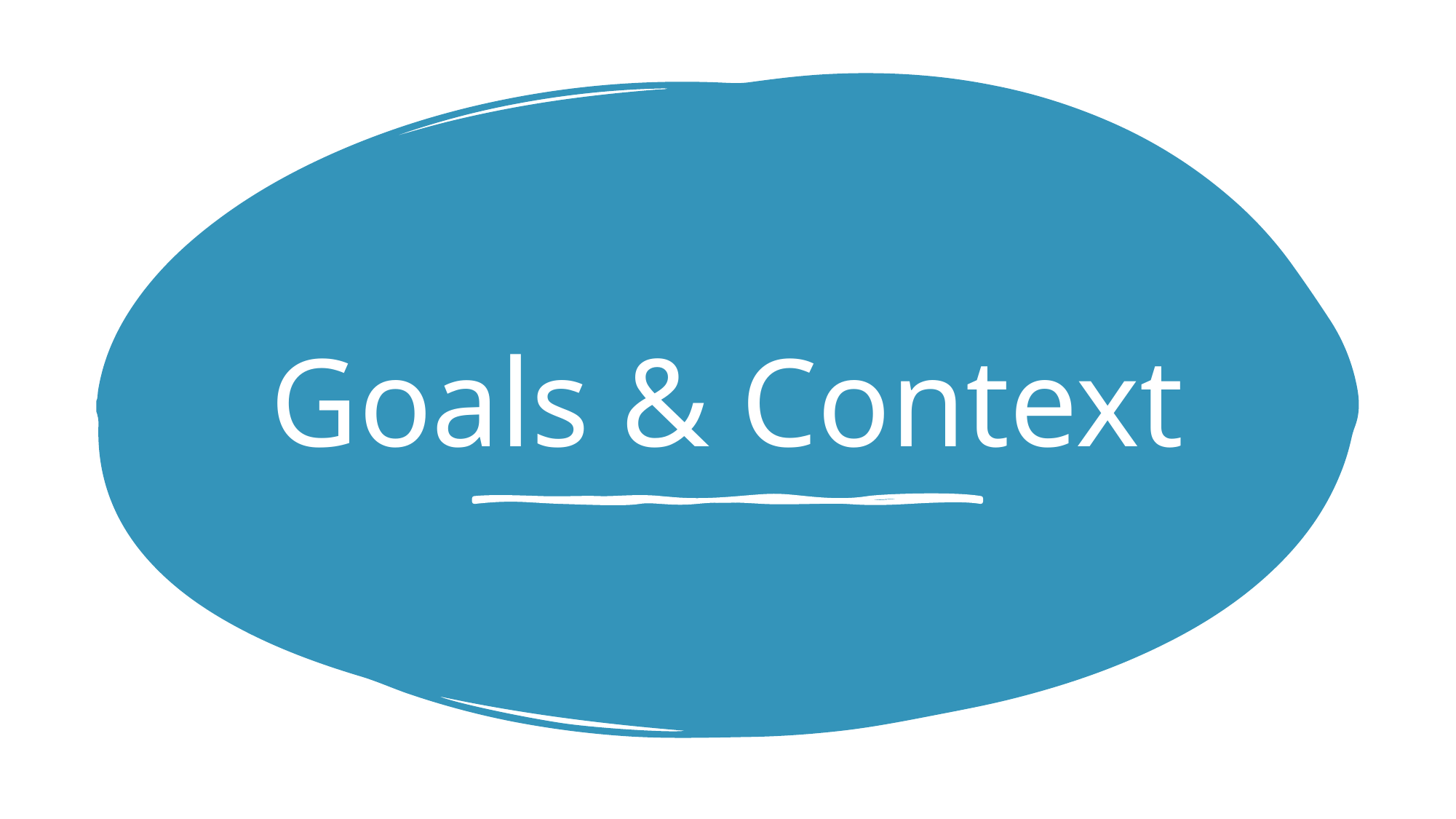

# Goals & Context

## Slide 5
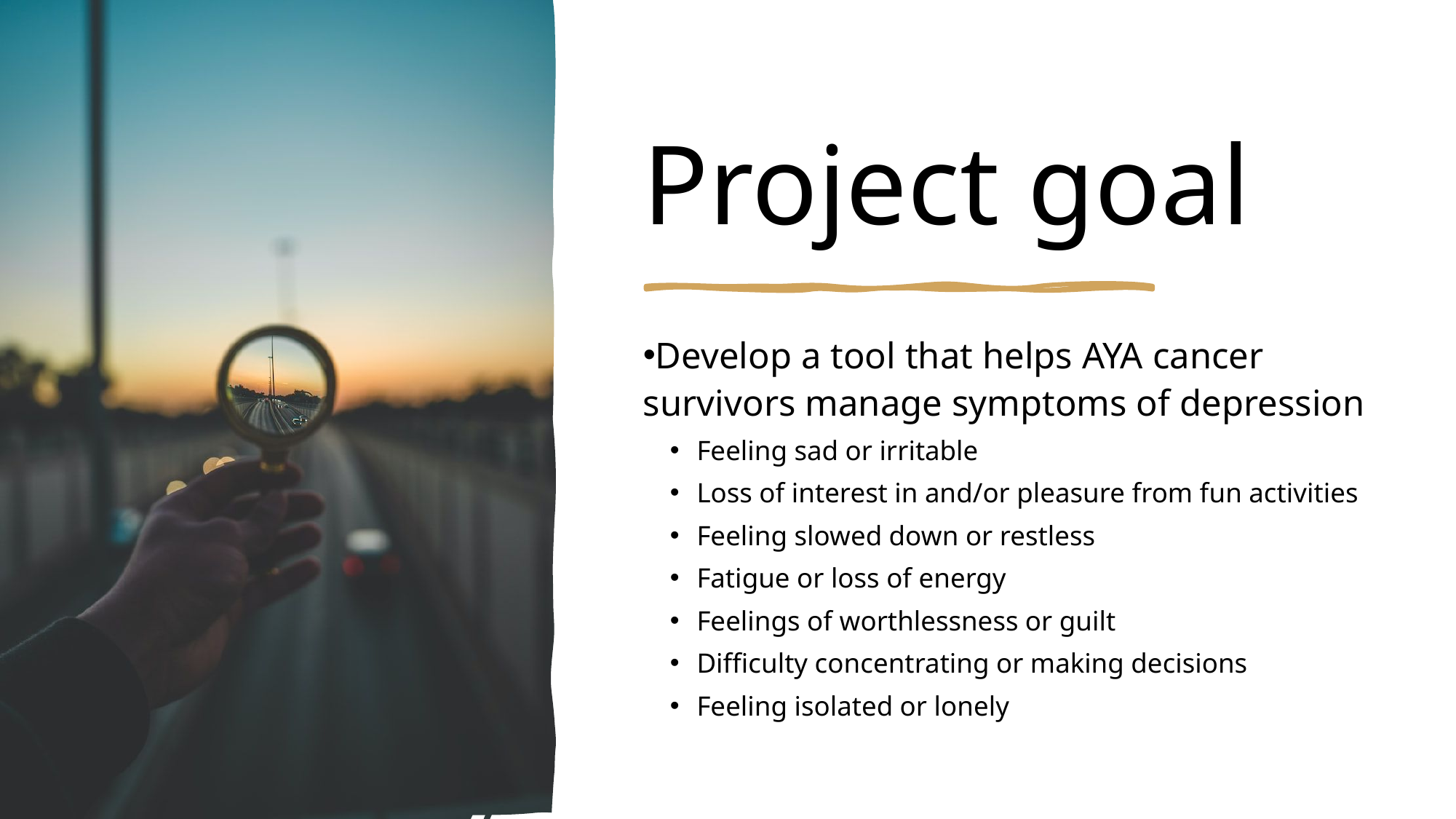

# Project goal
Develop a tool that helps AYA cancer survivors manage symptoms of depression
Feeling sad or irritable
Loss of interest in and/or pleasure from fun activities
Feeling slowed down or restless
Fatigue or loss of energy
Feelings of worthlessness or guilt
Difficulty concentrating or making decisions
Feeling isolated or lonely

## Slide 6
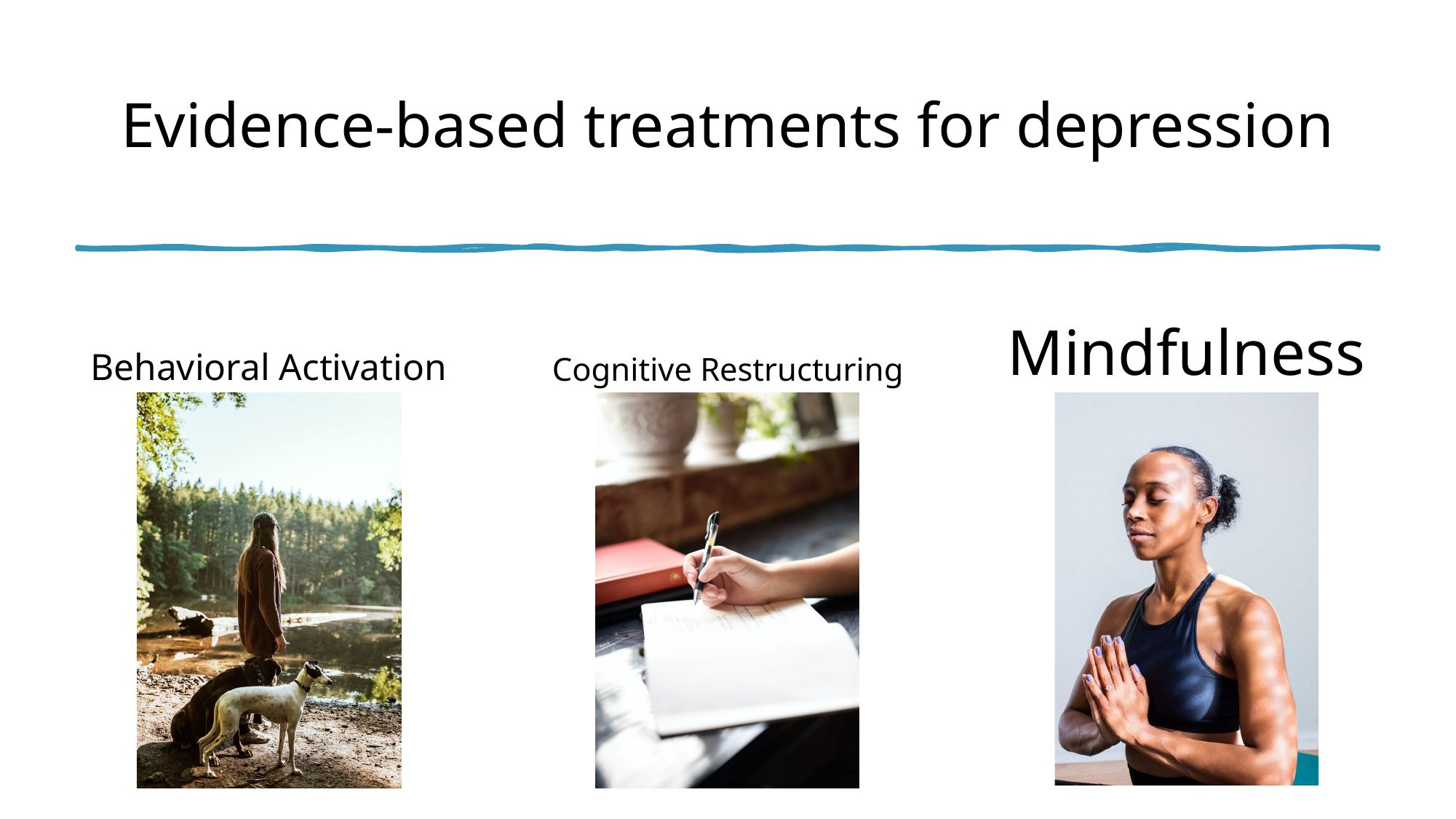

# Evidence-based treatments for depression
Behavioral Activation
Cognitive Restructuring
Mindfulness

## Slide 7
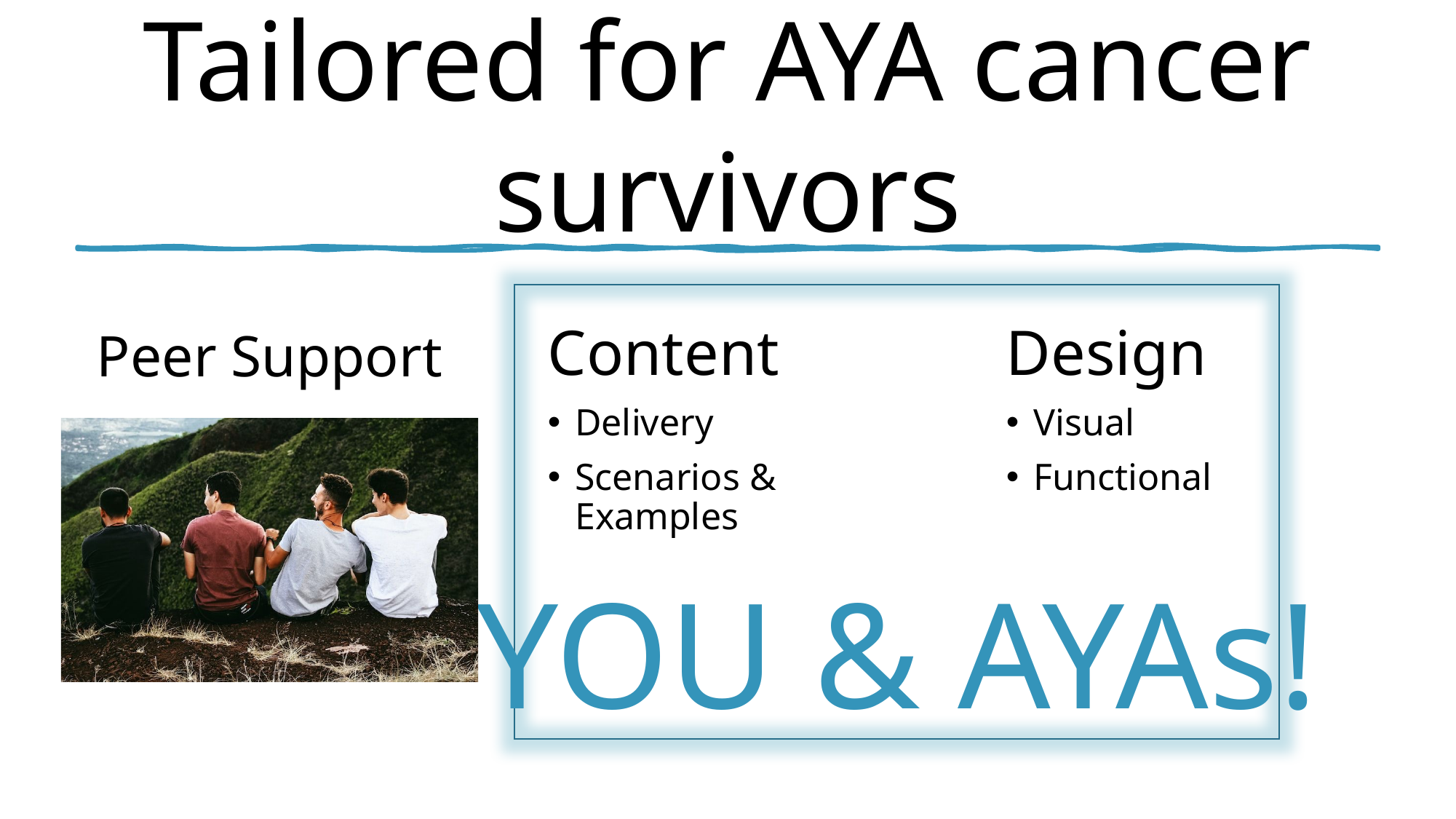

# Tailored for AYA cancer survivors
Content
Design
Peer Support
Delivery
Scenarios & Examples
Visual
Functional
YOU & AYAs!

## Slide 8
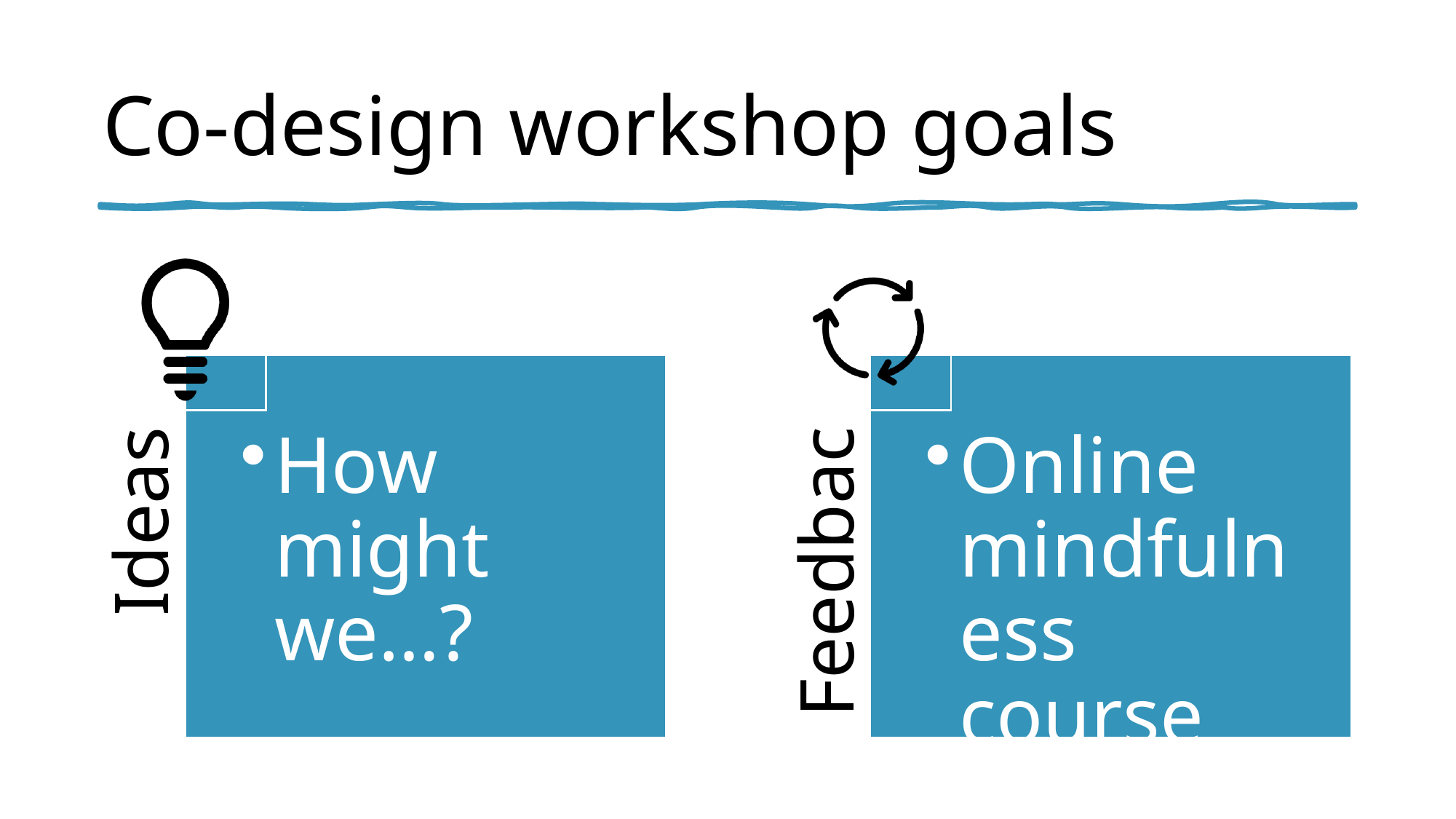

# Co-design workshop goals

## Slide 9
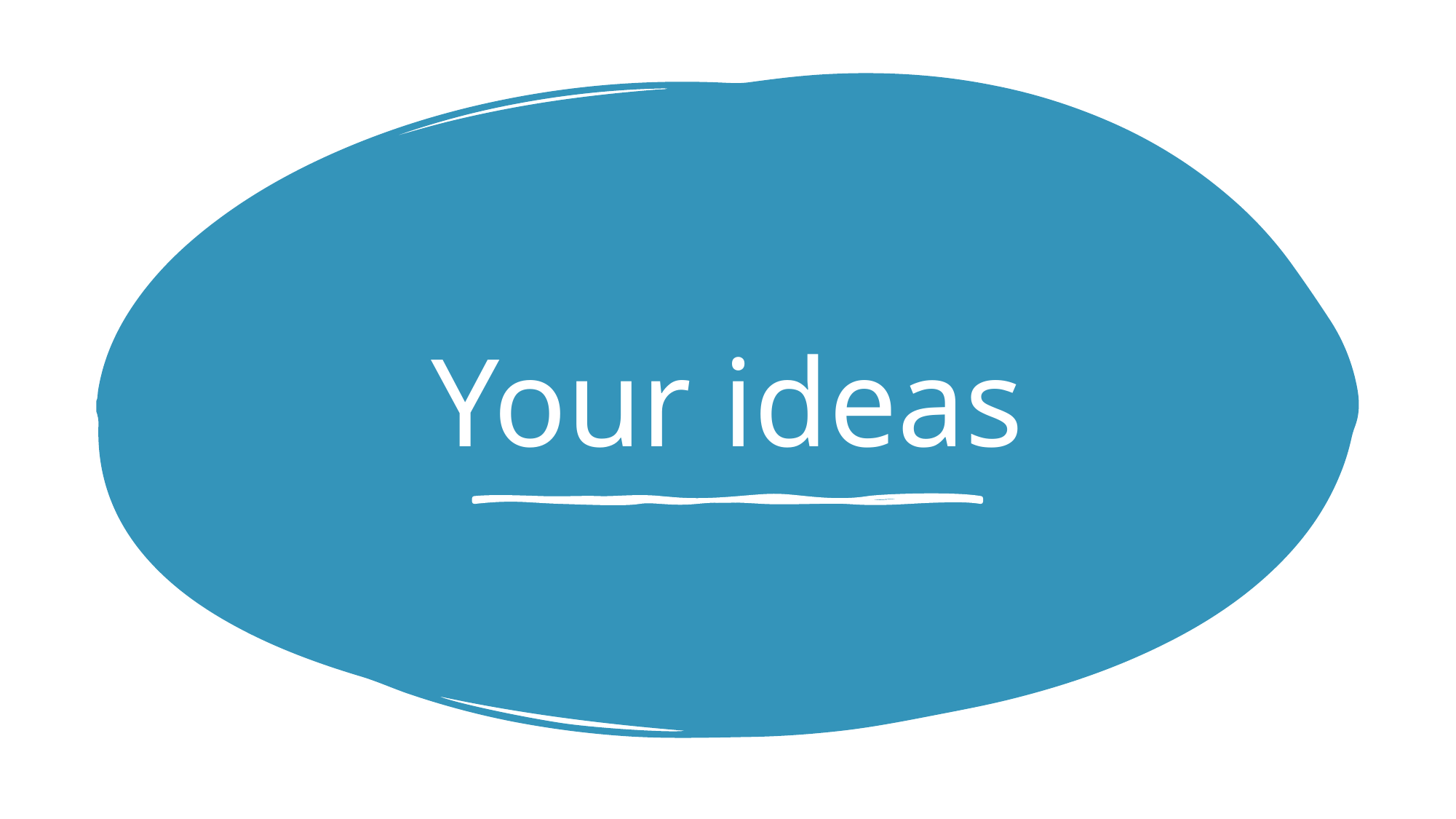

# Your ideas

## Slide 10
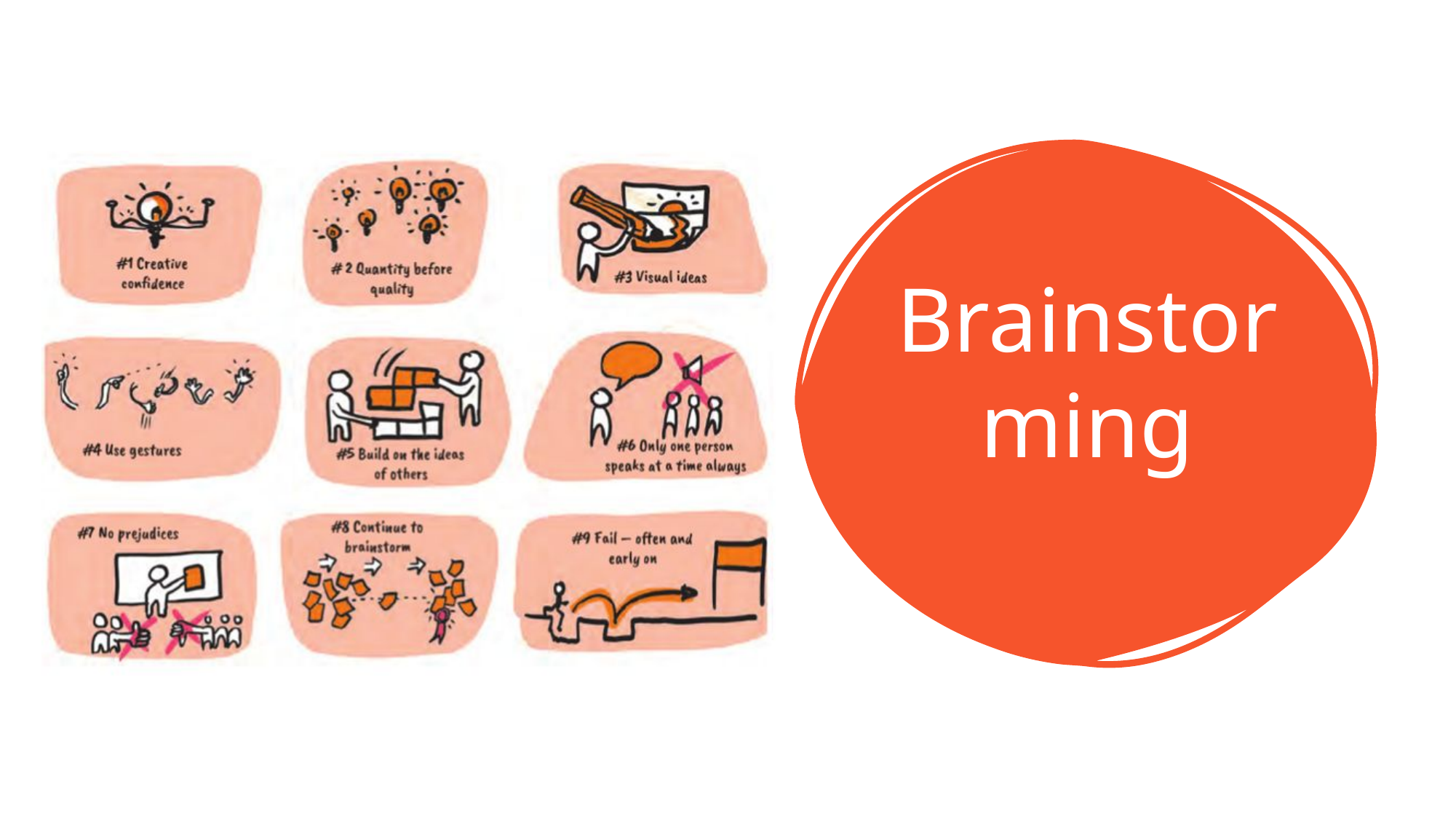

# Brainstorming

## Slide 11
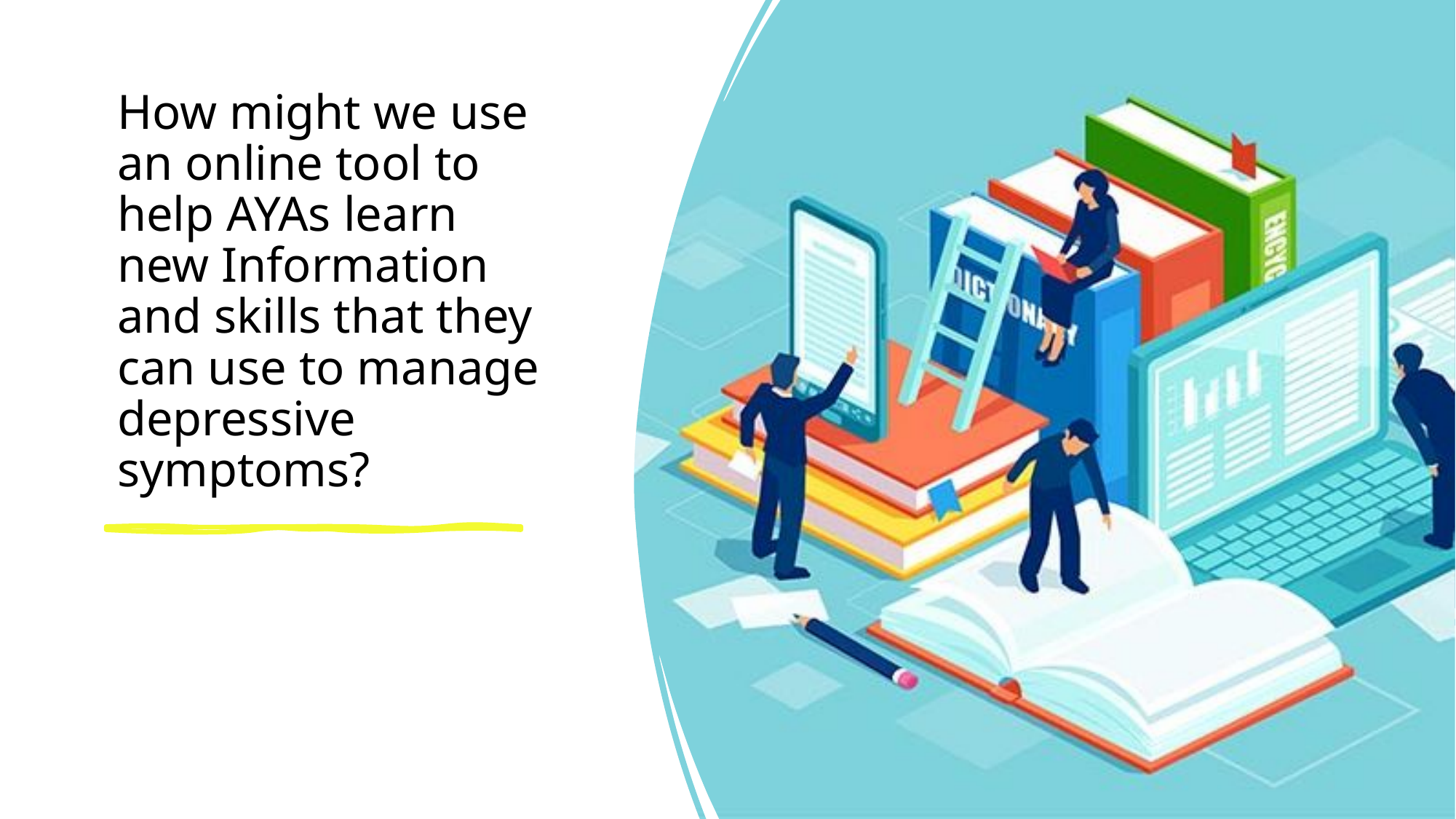

# How might we use an online tool to help AYAs learn new Information and skills that they can use to manage depressive symptoms?

## Slide 12
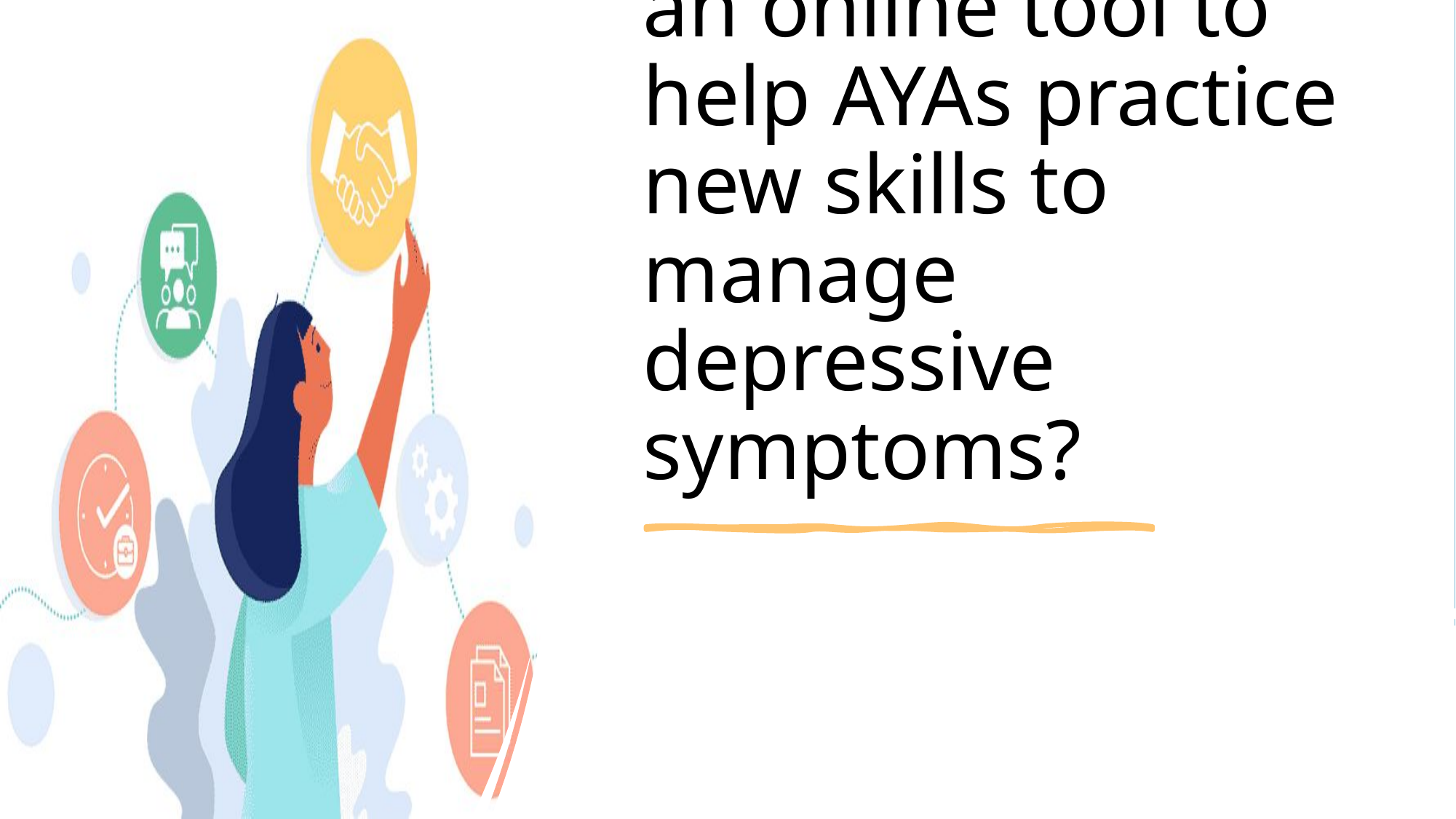

# How might we use an online tool to help AYAs practice new skills to manage depressive symptoms?

## Slide 13
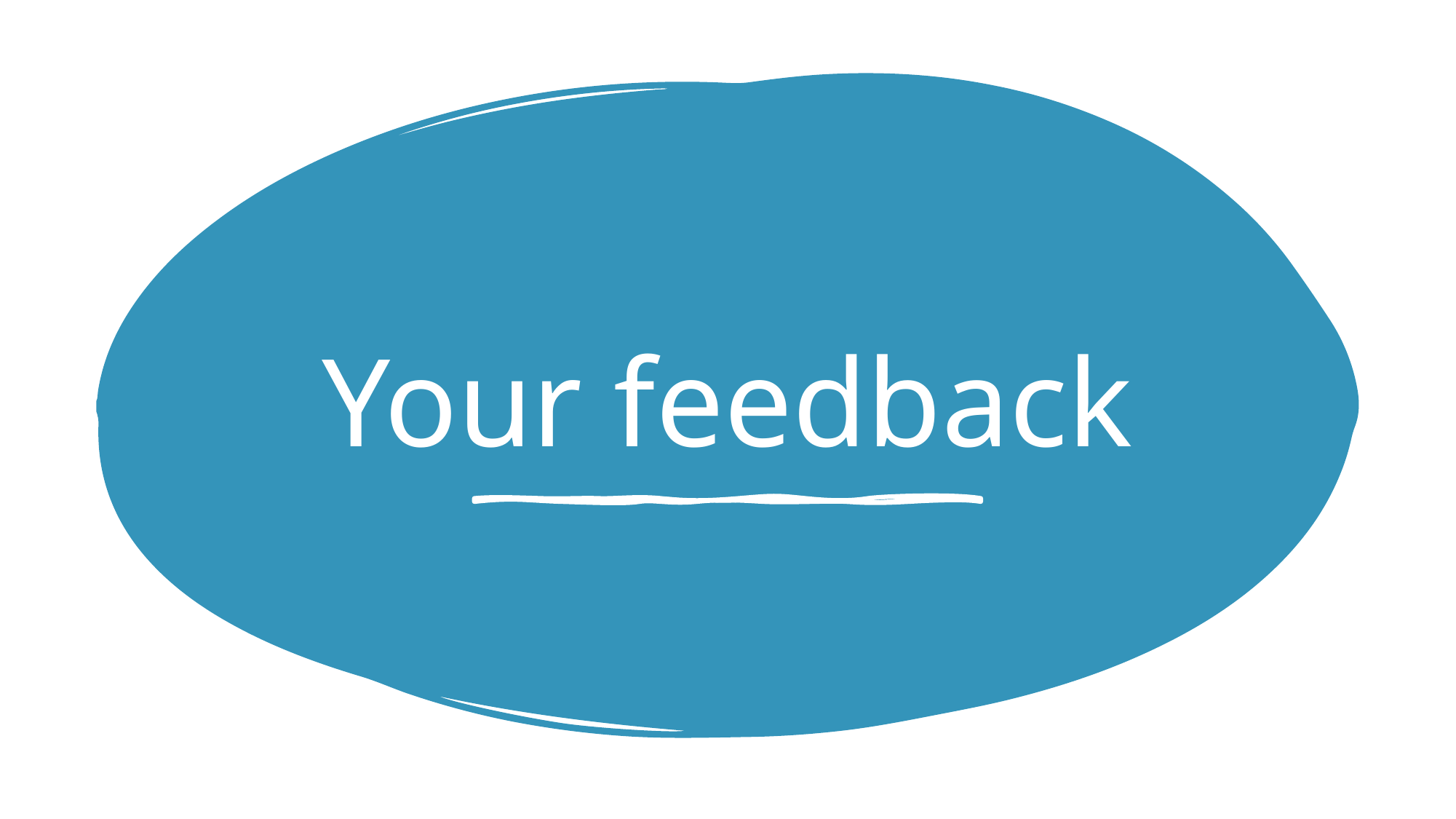

# Your feedback

## Slide 14
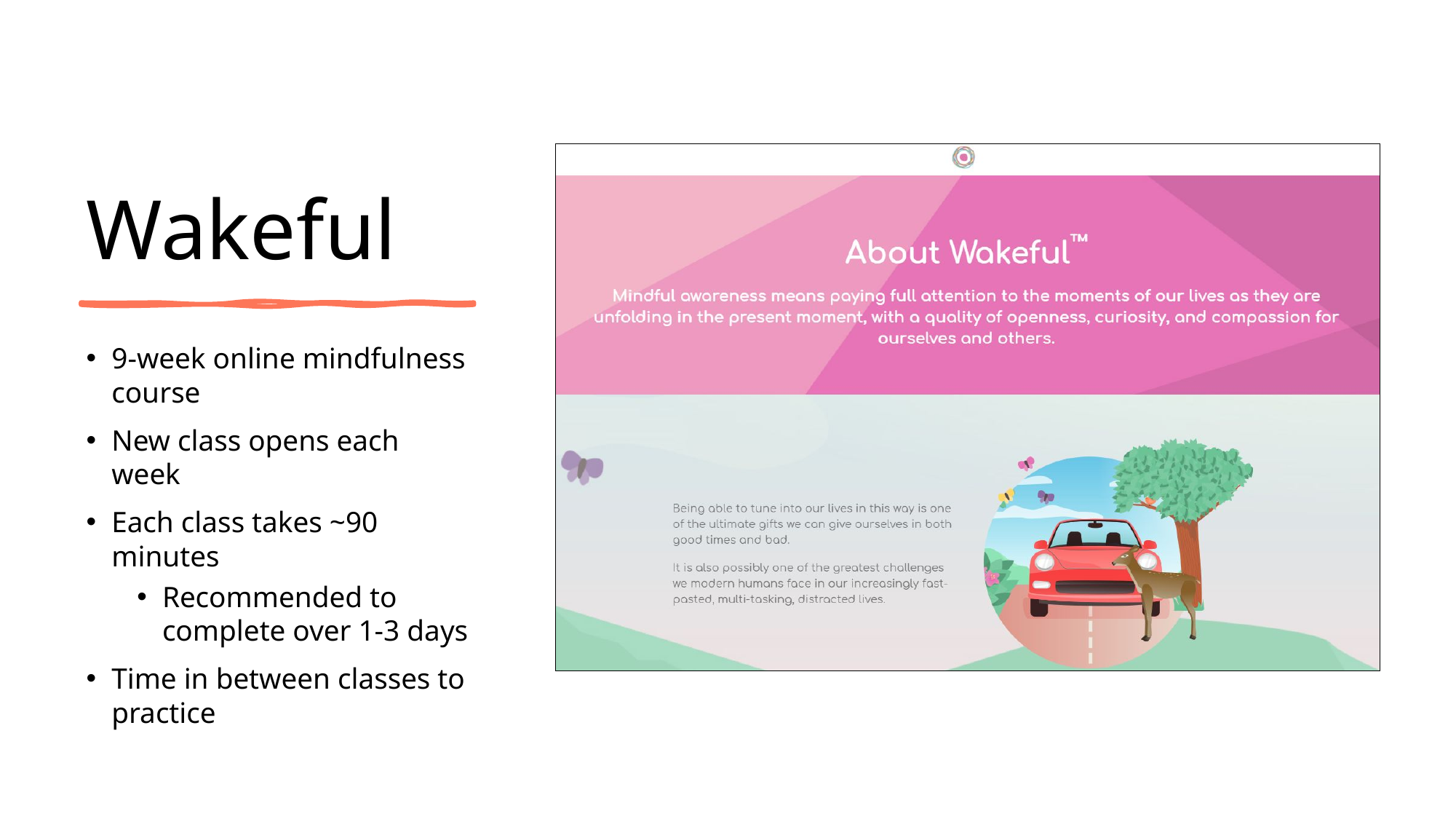

# Wakeful
9-week online mindfulness course
New class opens each week
Each class takes ~90 minutes
Recommended to complete over 1-3 days
Time in between classes to practice

## Slide 15
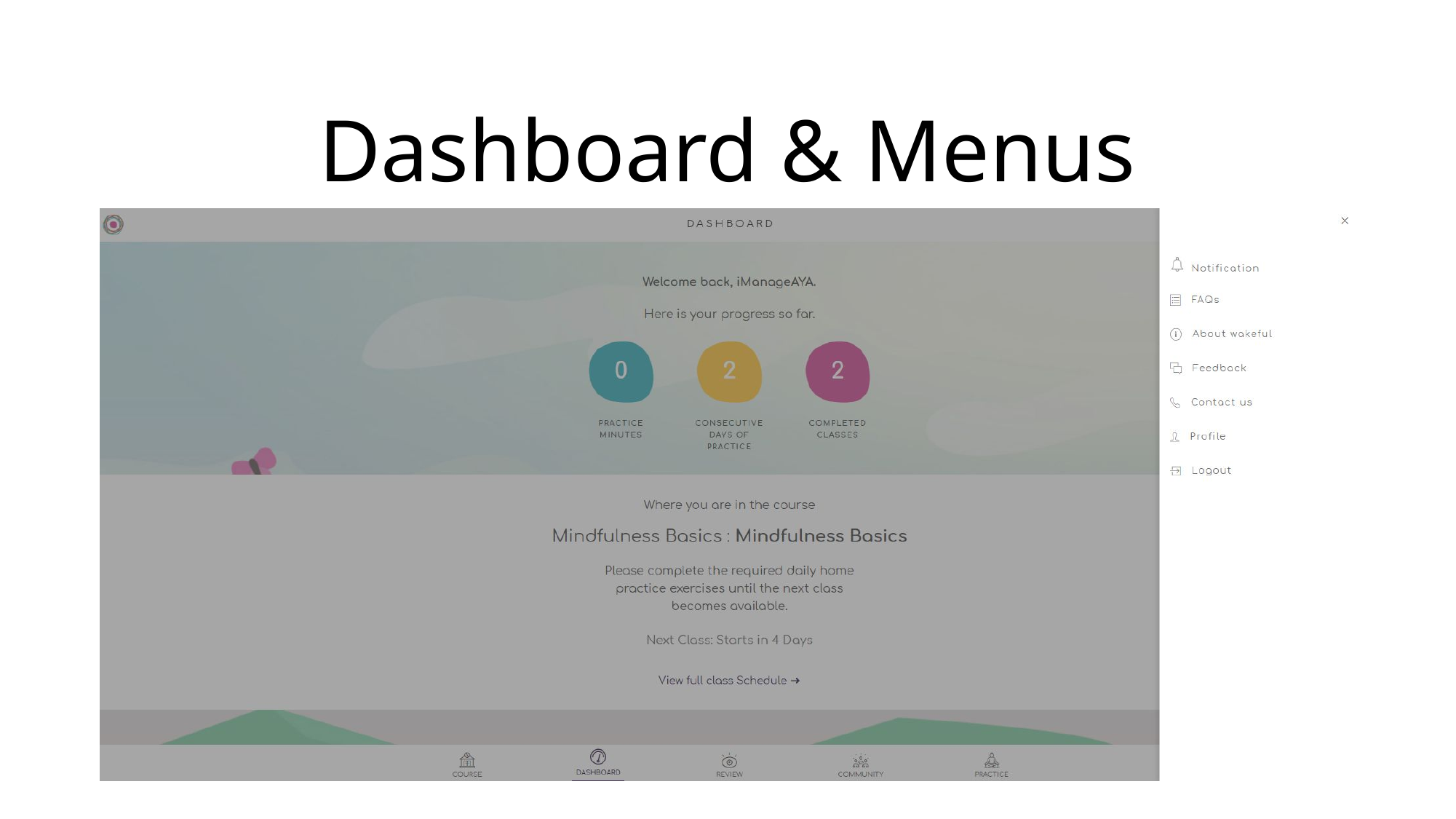

# Dashboard & Menus

## Slide 16
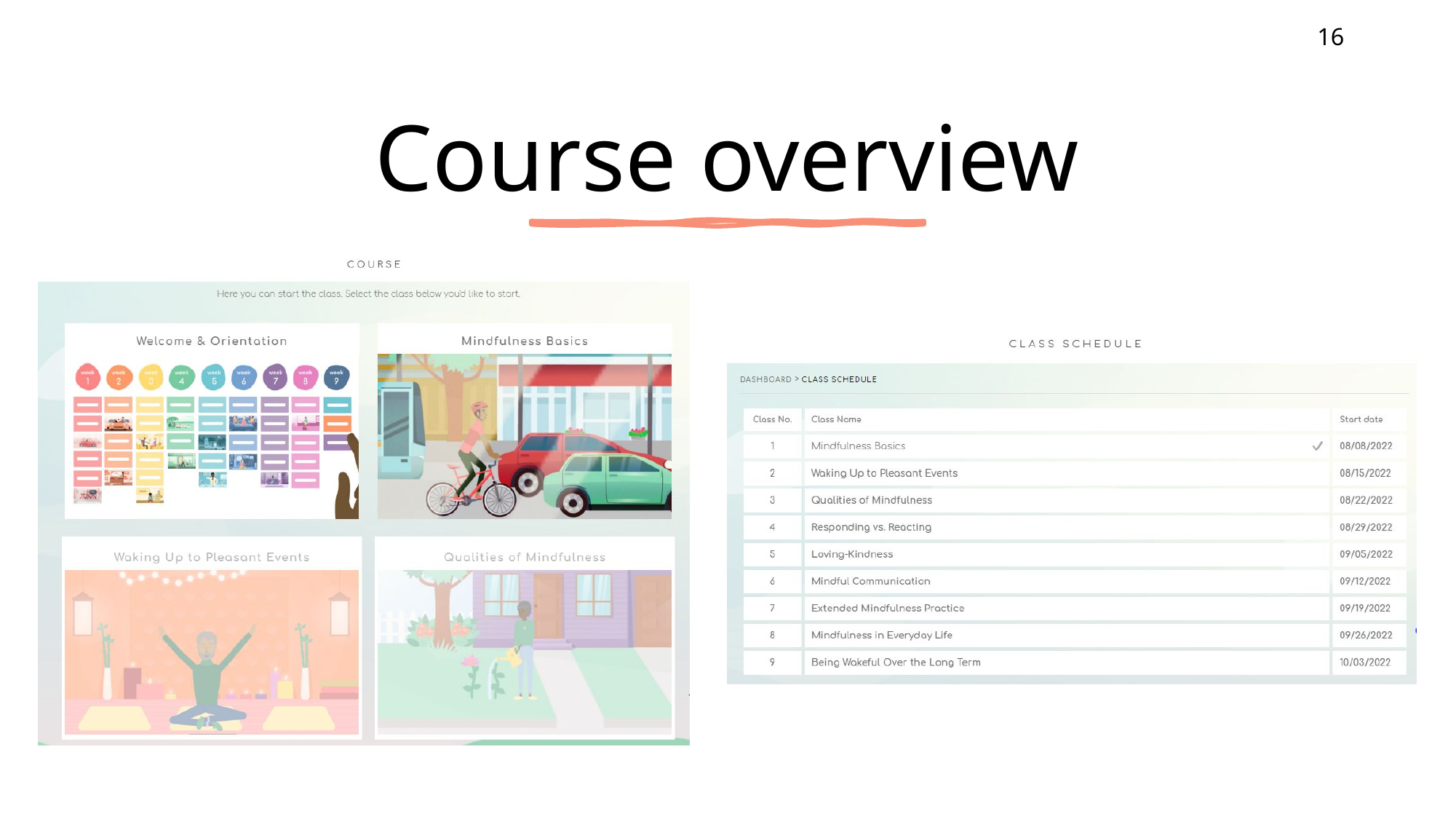

16
# Course overview

## Slide 17
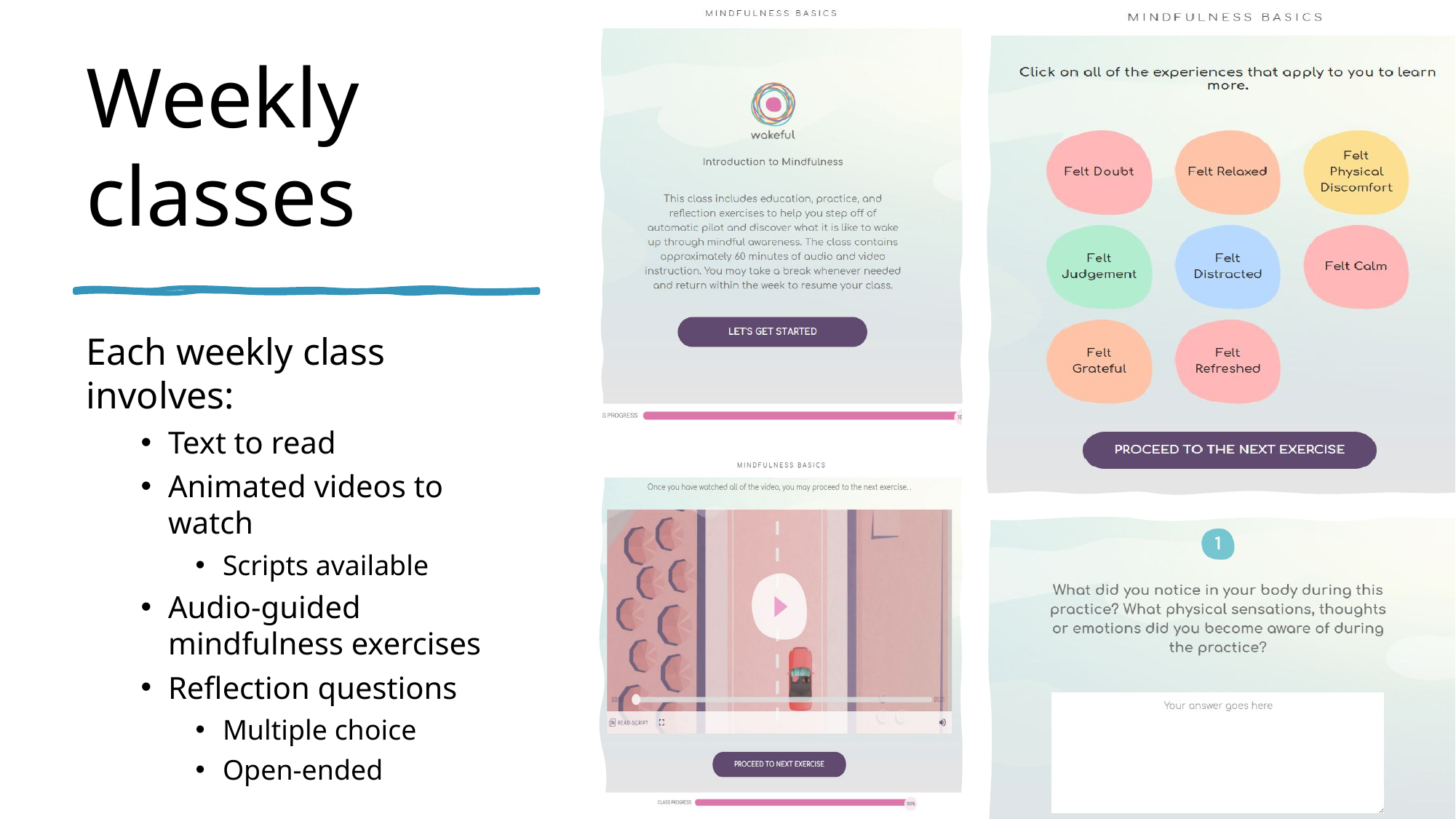

# Weekly classes
Each weekly class involves:
Text to read
Animated videos to watch
Scripts available
Audio-guided mindfulness exercises
Reflection questions
Multiple choice
Open-ended
17

## Slide 18
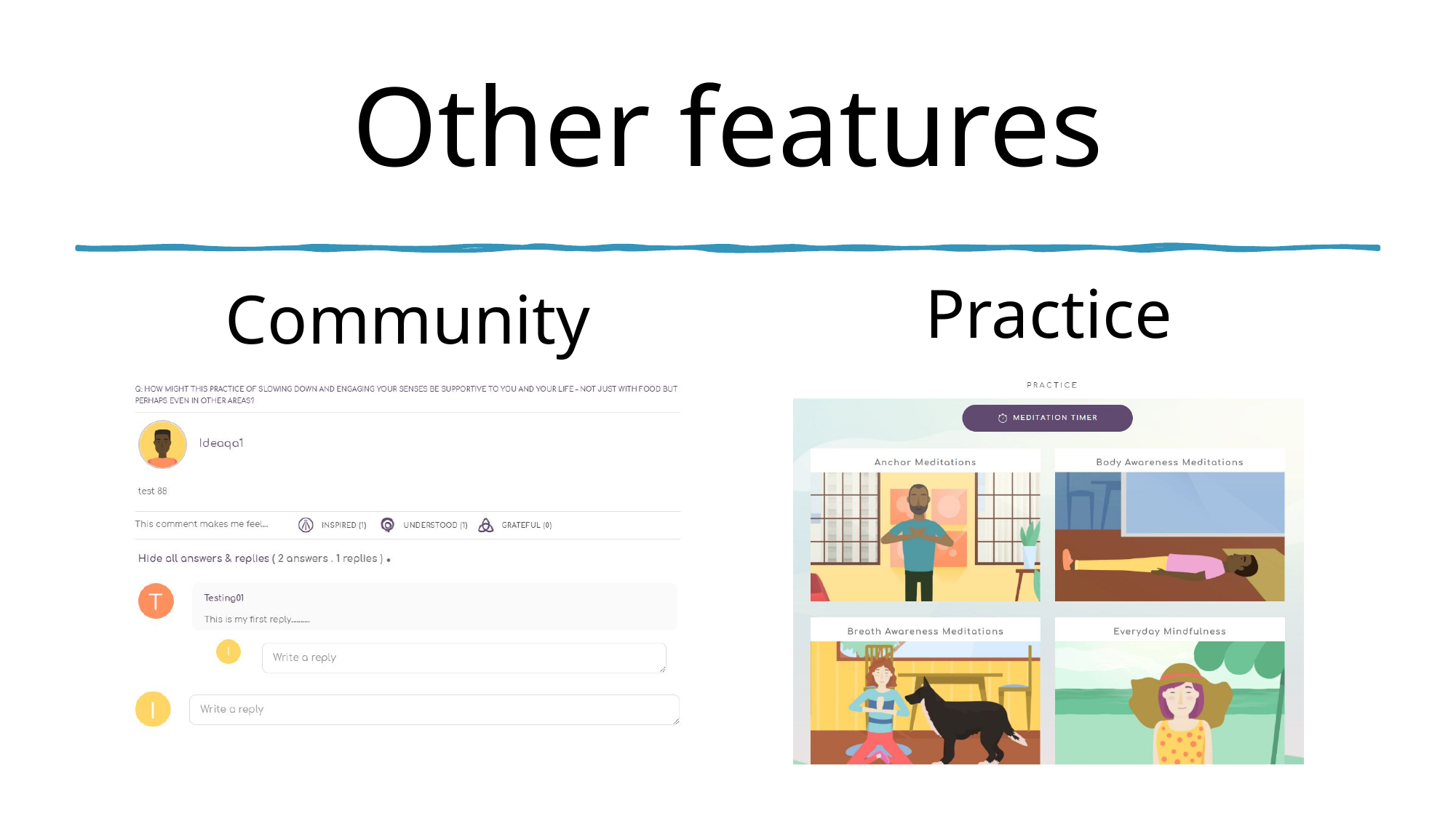

# Other features
Practice
Community

## Slide 19
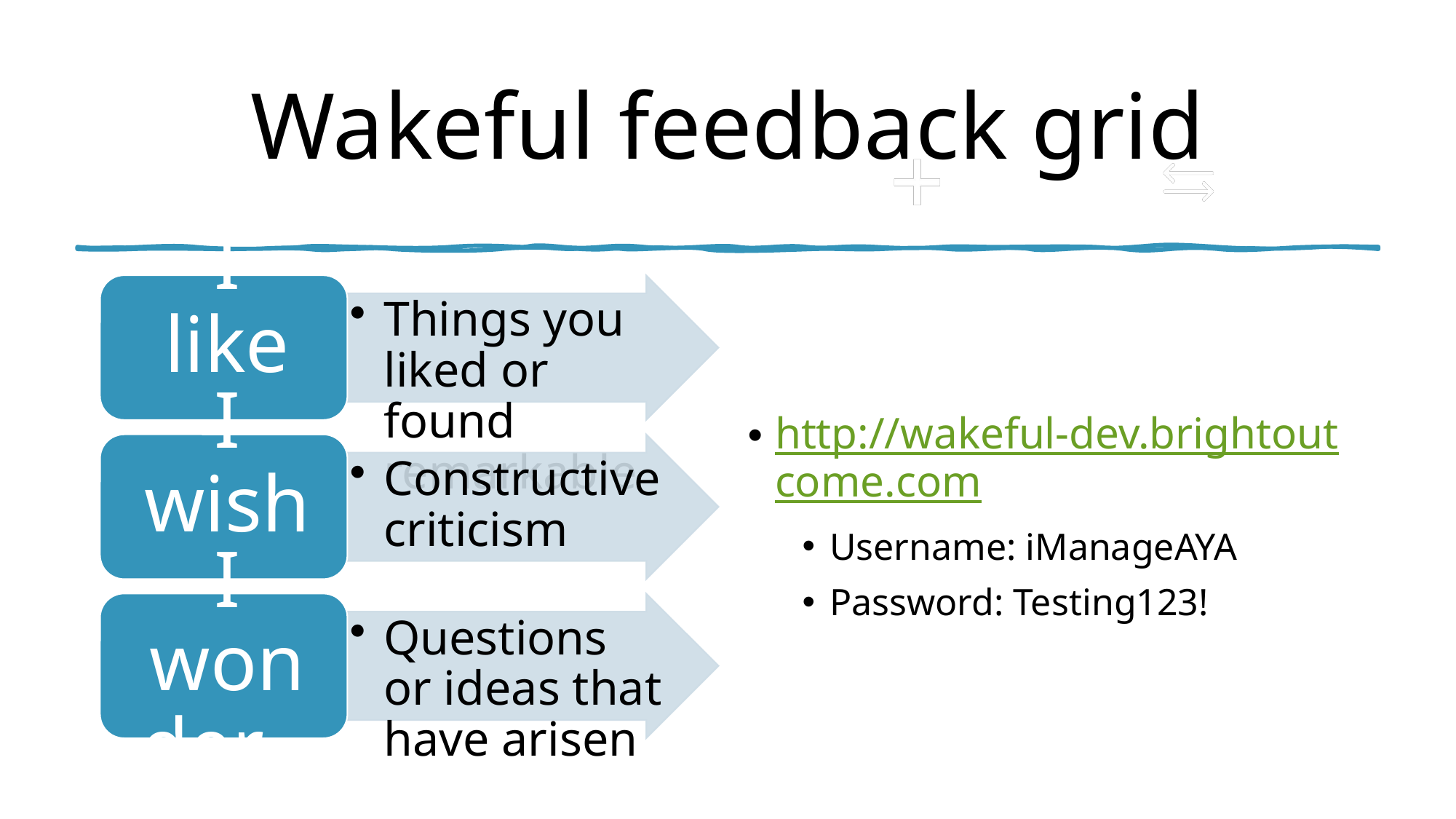

# Wakeful feedback grid
http://wakeful-dev.brightoutcome.com
Username: iManageAYA
Password: Testing123!

## Slide 20
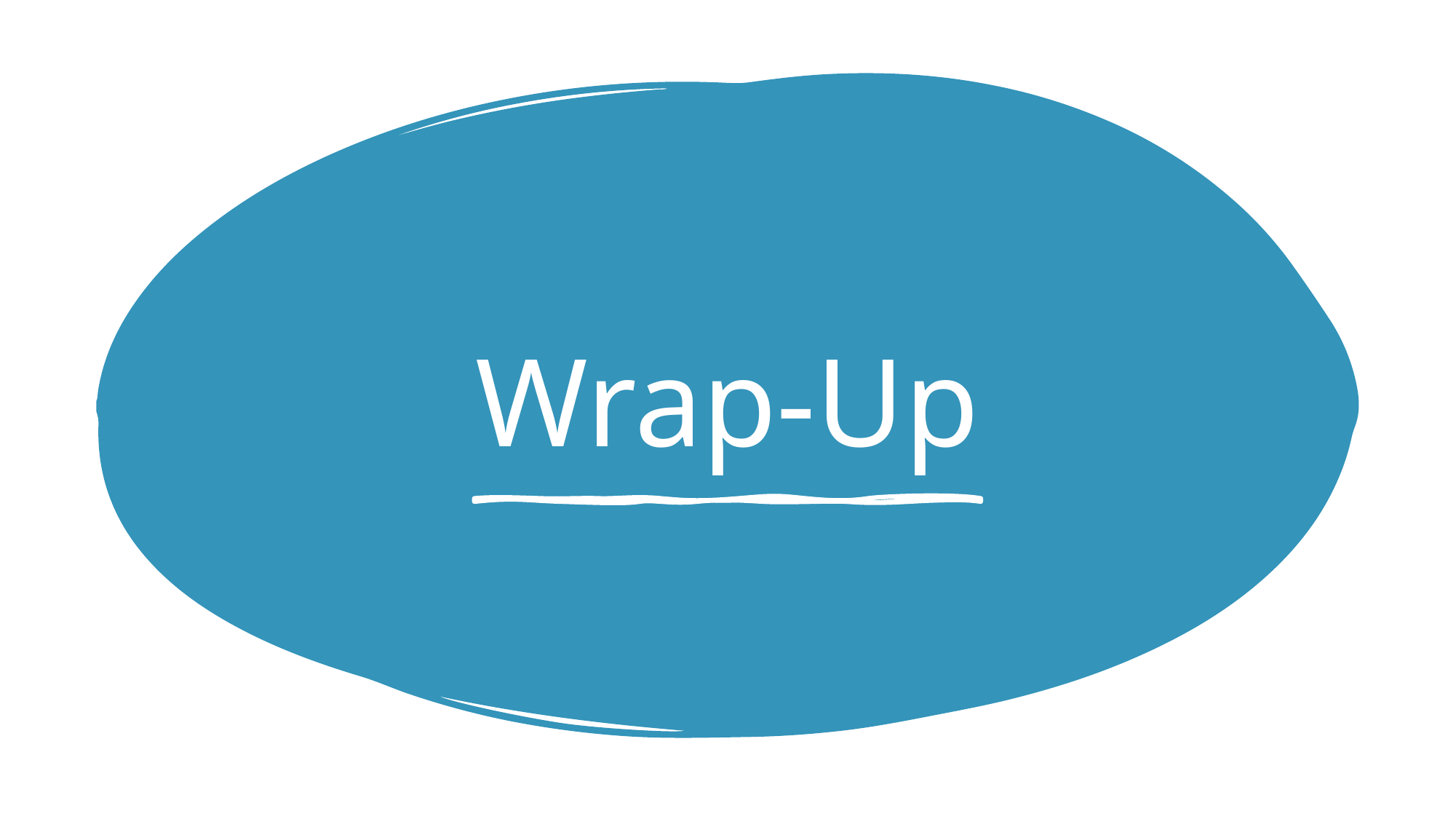

# Wrap-Up

## Slide 21
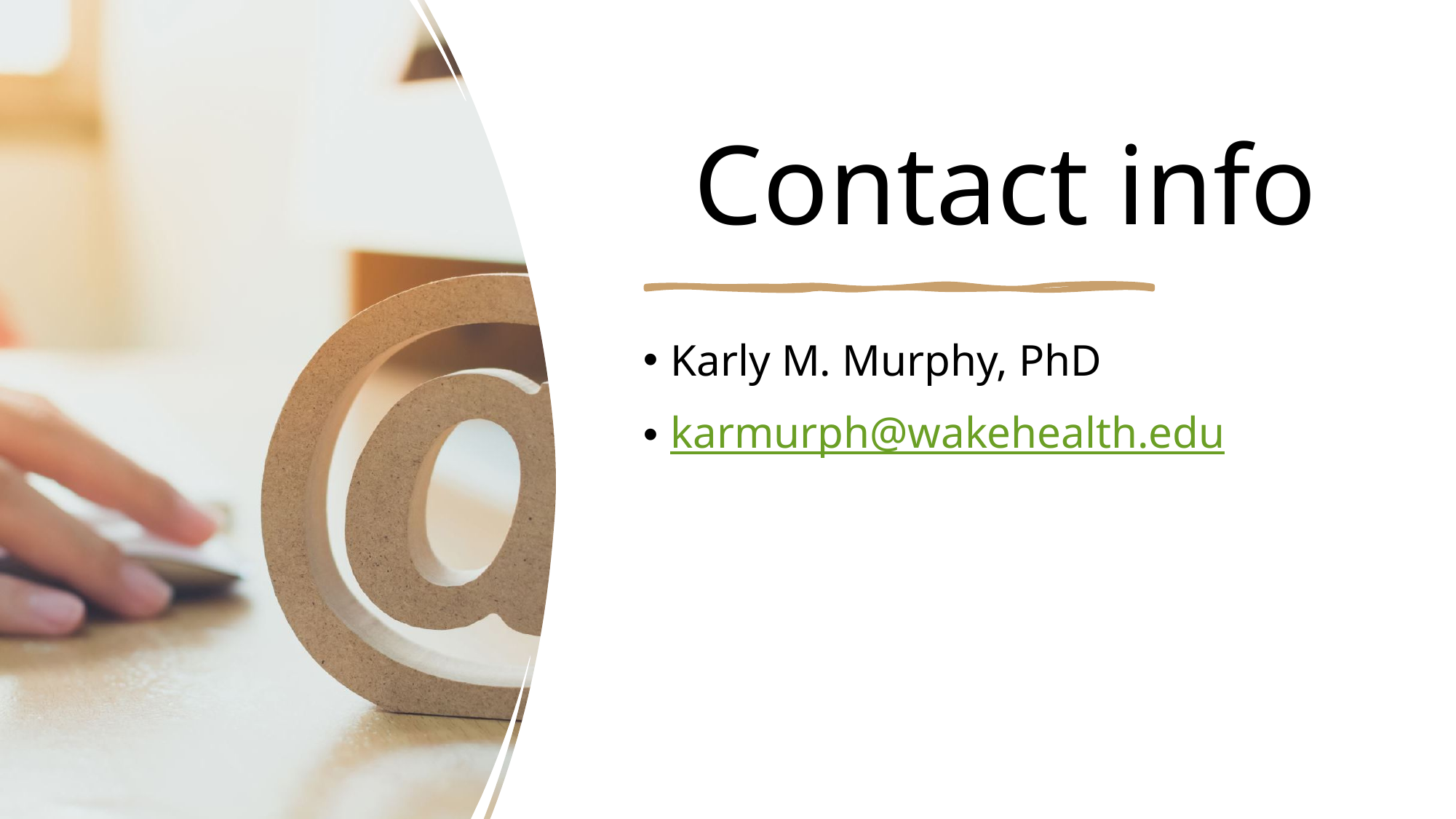

# Contact info
Karly M. Murphy, PhD
karmurph@wakehealth.edu
